# Hypercoagulability in critically ill patients with COVID 19, an observational prospective study

**DOI:** 10.1371/journal.pone.0277544

**Published:** 2022-11-23

**Authors:** Laure Calvet, François Thouy, Olivier Mascle, Anne-Françoise Sapin, Kévin Grapin, Jean Mathias Liteaudon, Bertrand Evrard, Benjamin Bonnet, Mireille Adda, Bertrand Souweine, Claire Dupuis

**Affiliations:** 1 Service de Médecine Intensive et Réanimation, CHU de Clermont-Ferrand, Clermont-Ferrand, France; 2 Laboratoire d’hématologie, CHU de Clermont-Ferrand, Clermont-Ferrand, France; 3 Service d’Immunologie, CHU Gabriel-Montpied, Clermont-Ferrand, France; 4 Laboratoire d’Immunologie, ECREIN, UMR1019 UNH, UFR Médecine de Clermont-Ferrand, Université Clermont Auvergne, Clermont-Ferrand, France; 5 Université Clermont Auvergne, CNRS, LMGE, Clermont-Ferrand, F-63000, France; 6 Université Clermont Auvergne, Unité de Nutrition Humaine, INRAe, CRNH Auvergne, Clermont-Ferrand, F-63000, France; Clinica Luganese Moncucco, SWITZERLAND

## Abstract

**Objective:**

COVID 19 is often associated with hypercoagulability and thromboembolic (TE) events. The aim of this study was to assess the characteristics of hypercoagulability and its relationship with new-onset TE events and the composite outcome of need for intubation and/or death in intensive care unit (ICU) patients admitted for COVID.

**Design:**

Prospective observational study.

**Setting:**

Monocentric, intensive care, University Hospital of Clermont Ferrand, France.

**Patients:**

Patients admitted to intensive care from January 2020 to May 2021 for COVID-19 pneumonia.

**Interventions:**

Standard hemostatic tests and rotational thromboelastometry (ROTEM) were performed on admission and on day 4. Hypercoagulability was defined by at least one of the following criteria: D-dimers > 3000 μg/dL, fibrinogen > 8 g/L, EXTEM CFT below the normal range, EXTEM A5, MCF, Li 60 above the normal range, and EXTEM G-score ((5000 x MCF) / (100-MCF)) ≥ 11 dyne/cm^2^.

**Measurements and main results:**

Of the 133 patients included, 17 (12.7%) developed new-onset TE events, and 59 (44.3%) required intubation and/or died in the ICU. ROTEM was performed in 133 patients on day 1 and in 67 on day 4. Hypercoagulability was present on day 1 in 115 (86.4%) patients. None of the hypercoagulability indices were associated with subsequent new-onset TE events on days 1 and 4 nor with the need for intubation and/or ICU death. Hyperfibrinogenemia > 8g/dL, higher D-dimers and higher EXTEM Li 60 on day 4 were predictive of need for intubation and/or of ICU death.

**Conclusions:**

Our study confirmed that most COVID-19 ICU patients have hypercoagulability on admission and almost all on day 4. Hyperfibrinogenemia or fibrinolysis shutdown on day 4 were associated with unfavorable outcome.

## Introduction

Patients with severe forms of COVID 19 have multifactorial hypercoagulability: increased D-dimer, fibrinogen, factor VIII levels, decreased protein C, protein S and antithrombin levels, platelet hyperaggregability, endothelial damage by Sars Cov 2 [1–3], and hypofibrinolysis. This hypercoagulability leads to thromboembolic events (TE) concerning macro and micro circulation [4–7]. These TE events, notably microcirculation thrombosis and pulmonary embolism, represent one of the leading causes of multiple organ failure in critically ill COVID-19 patients [4,6,8–14]. Thus, high dose preventive anticoagulation (HDPA) or even curative dose anticoagulation (CDA) were initially recommended in patients with severe forms of COVID-19 admitted to intensive care units [15–17].

In patients with COVID-19, several studies have reported that standard coagulation tests (SCT), such as platelet count, prothrombin time (PT), activated partial thromboplastin time (aPTT), and international normalized ratio (INR), may show normal results despite the presence of hypercoagulability [10]. Additionally, PT, aPTT, D-dimer and plasma fibrinogen assays do not explore fibrinolysis.

Rotational thromboelastometry (ROTEM) is a relocated laboratory assay providing a comprehensive analysis of dynamic and timely changes in the viscoelastic properties of clot formation, including clot initiation, propagation, strengthening, and dissolution. ROTEM tests assess both coagulation and fibrinolysis, are performed on whole blood and therefore take into account the contribution of blood cells. In ROTEM tests, clot formation can be triggered in various ways by the addition of different activators, defining different assays: EXTEM to assess the extrinsic pathway, INTEM the intrinsic pathway, FIBTEM the effect of fibrinogen on coagulation, and HEPTEM that of heparin on coagulation. In ROTEM assays, a probe is suspended within a sample of whole citrated blood, and changes in blood viscosity, which develop while the sample is rotated, are graphically transmitted. (S1 File).

ROTEM is recommended to guide the transfusion strategy in the management of major bleeding, since it can reduce blood product consumption [18–20]. It can also be performed to assess hypercoagulability [21–24], predict thrombosis in subgroups of patients at increased risk of TE events [25–28], and identify the effect of heparin on ROTEM results using heparinase (HEPTEM) [29]. Several studies performed on ROTEM in ICU patients admitted for COVID reported hypercoagulability [30] and hypofibrinolysis [31–33] in patients with and without anticoagulant treatment that were present from the early phase of the disease [34], more severe in more acutely ill patients [35], and associated with unfavorable outcome [36].

Although several studies have attempted to establish a clear relationship between hypercoagulability and TE events and poor outcome in COVID-patients, such an association is still debated because of the small sample size of most studies, and variability in definitions of the procoagulant profile and of the clinical outcome assessment.

The aim of this study was to determine in critically ill patients admitted for COVID the prevalence of hypercoagulability on ICU admission and on day 4, compare the performance of standard coagulation and ROTEM tests for predicting TE events, and the unfavorable outcomes including need for intubation, ICU death, and the composite end point of need for intubation and/or ICU mortality.

## Methods

### Study population

This single center, prospective observational study was performed in the medical intensive care unit (ICU) of the university hospital of Clermont-Ferrand, France. All consecutive adult patients admitted between April 1, 2020 and March 31, 2021 for acute hypoxemic respiratory failure with a positive SARS COV 2 polymerase chain reaction (PCR)-based technique were included. CT pulmonary angiography was systematically performed on ICU admission. CT angiography (pulmonary/abdomino-pelvic/lower limbs/brain) was performed during ICU stay according to the evolution of clinical or laboratory parameters suggestive of TE events. TE events diagnosed before ICU admission or during ICU stay (new-onset TE event) were recorded. Patients were given antithrombotic treatment with LMWH or UFH using standard dose prophylactic anticoagulation (SPA), high dose prophylactic anticoagulation (HDPA), and curative dose anticoagulation (CDA) (S1 File) in accordance with the recommendations of the ’Groupe d’Etude sur l’Hémostase et la Thrombose’(GEHT) [15]. Demographic and clinical information, and laboratory and imaging data were extracted from the electronic charts. Vital status on ICU and hospital discharge were also recorded. Patients were managed without specific therapeutic intervention.

### Ethical considerations

All patients or their relatives received fair and relevant information. They gave written informed consent for the storage and research use of residual blood from samples collected as part of routine care.The study was approved by the ethics committee of the « Société de Réanimation de Langue Française » (SRLF) (CE SRLF n° 20–96) (CE French acronym for “commission d’éthique”). All the procedures were followed in accordance with the ethical standards of the responsible committee on human experimentation (institutional or regional) and with the Helsinki Declaration of 1975.

### Blood samples and laboratory features

Blood count and standard coagulation tests were performed daily during ICU stay including platelet count, activated partial thromboplastin time (aPTT) prothrombin time (PT), fibrinogen, and D-dimer (S1 File). On day 1, blood values of inflammatory parameters, including C-reactive protein (CRP), ferritin, interleukin-1 (IL1), IL-6, IL10, and monocyte HLA-DR (mHLA-DR) expression were measured as previously reported [37]. No patients received tocilizumab before day 1.

On day 1 and day 4 ± 1, two milliliters of residual blood from a citrate-containing tube were used to perform ROTEM tests in the ICU with a point-of-care testing device (ROTEM sigma, Werfen, Le pré saint Gervais, France).

The ROTEM tests, including extrinsic (EXTEM), intrinsic (INTEM), functional fibrinogen (FIBTEM), heparinase (HEPTEM) assays, were carried out in accordance with the manufacturer’s instructions. The following ROTEM parameters were analyzed: clotting time (CT, s), clot formation time (CFT, s), α-angle and amplitude at 5 minutes (A5, mm), maximum clot firmness, (MCF, mm), and lysis index, the percentage of the remaining clot firmness in percentage of MCF at 60 minutes after CT (Li60, %).

### Definitions

TE events were defined by the onset of one of the following events: deep venous thrombosis (DVT), pulmonary embolism (PE), either catheter-related or not, ischemic stroke, systemic arterial thromboembolism, myocardial infarction, circuit clotting during renal replacement therapy (RRT), thrombosis in the extracorporeal membrane oxygenation (ECMO) circuit, diagnosed by ultrasound, computed tomography scan and ECG/Troponin, and clotting of the extracorporeal circuit.

Hypercoagulability was defined by at least one of the following indices: fibrinogen > 8 g/L, D-dimers > 3000 μg/dL, EXTEM CFT shorter than normal range, EXTEM A5 higher than normal range, EXTEM MCF higher than normal range, G score ≥11 dyne/cm^2^, and EXTEM Li 60 > 96.5%. The EXTEM G-score was defined as follows: G-score = (5000xMCF) / (100-MCF). A major role of fibrinogen in a prothrombotic state was defined by a greater difference between observed and normal MCF values of FIBTEM than between those of EXTEM. Heparin was considered to affect ROTEM results when INTEM CT/ HEPTEM CT ratio was higher than 1.

Major bleeding events were recorded according to ISTH guidelines [38] (S1 File).

### Statistical analysis

Patient characteristics were expressed as n (%) for categorical variables and median (interquartile range [IQR]) for continuous variables. Comparisons were made with Student or Mann-Whitney tests and the Chi2 or Fisher exact tests for continuous and categorical data, respectively, depending on the distribution of the data. The correlations between all coagulation indices on admission were explored using coefficient correlations of Pearson or Spearman as appropriate.

Indices on admission and on day 4 were compared with paired t test or Wilcoxon test for paired data for continuous variables, and with McNemar’s test for categorical data.

Factors associated with (1) the occurrence of TE events among patients free of TE events on admission, (2) intubation (excluding patients who died without intubation because of withholding of life support), (3) ICU death, and (4) the composite outcome including ICU death and/or intubation were investigated using Student or Wilcoxon tests, and the Chi2 or Fisher exact tests for continuous and categorical data, respectively, depending on the distribution of the data.

The ability of the coagulation indices to predict (1) TE events, (2) intubation, (3) ICU death, and (4) ICU death and/or intubation was assessed using the ROC curve. The best threshold value for the continuous variables was determined, and their corresponding area under the curve, sensibility and specificity were recorded.

A sensitivity analysis for missing data was performed to determine the amount and statistical nature of the missing data (informative and non-informative missing data: Missing At Random, Missing Completely At Random, Not Missing At Random). Missing data were imputed using multiple imputation if needed.

A p value of < 0.05 was considered statistically significant. SAS® (Version 9.4; SAS Institute, Cary, NC, USA) and R (Version 3.4.0; R Core Team, Wien, Austria) softwares were used for the analysis.

## Results

### Baseline characteristics

During the study period, 133 patients were enrolled in the study (Table 1). In 23 patients, the medical background included TE events. In 10 patients, 7 PEs and 3 DVTs were diagnosed on ICU admission. In 17 (12.8%) patients, 21 new-onset TE events occurred during ICU stay (6 PEs, 2 DVTs, 1 catheter thrombosis, 1 acute lower limb ischemia, and 11 episodes of RRT filter clotting). Invasive mechanical ventilation was administered to 14 (10.6%) patients on day 1 and subsequently to 40 (31.2%) additional patients. ICU mortality rate was 24.8%. Ultimately, the composite outcome of intubation and/or ICU death was met by 59 (44.3%) patients.

**Table 1:** Characteristics of patients on admission and during ICU stay.

Baseline Characteristics	All, n = 133
Period of admission	Median(IQR), n(%)
March-August 2020	4 (3)
September- December 2020	75 (56.4)
January-June 2021	54 (40.6)
Age, median (IQR)	71.2 [63.4; 76.4]
Gender (Male)	95 (71.4)
BMI > 30 (kg/m^2^)	58 (43.6)
**Comorbidities**	
At least one	73 (57.4)
Diabetes	18 (13.6)
Cardiovascular disorder	31 (23.4)
Lung disease	8 (6)
Renal disease	16 (12)
Immunosuppression§	19 (14.2)
Thromboembolic disease	23 (17.2)
Time from onset of symptoms and ICU admission, days (missing = 12)	8 [5; 10]
Immunomodulatory treatments before ICU admission§§	11 (8.3)
Anticoagulation before ICU admission	
None	63 (47.4)
Prophylactic anticoagulation	16 (12)
High-dose prophylactic anticoagulation*	16 (12)
Curative anticoagulation	38 (28.6)
Anti-platelet therapy before ICU admission	36 (27)
**Severity on admission**	
SAPS II on admission	35 [29; 44]
SOFA score	4 [3; 6]
PaO2/FiO2	135 [116.6; 186]
IMV	14 (10.6)
HFNC	67 (50.4)
Thromboembolic event on admission	10 (7.6)
**Other treatments on days 1 or 2**	
Vasopressors	17 (12.8)
Renal replacement therapy	3 (2.2)
Steroids on admission	106 (79.6)
**Main events during ICU stay**	
IMV	40 (31.2)
Vasopressors	43 (33.6)
Renal replacement therapy	18 (14)
Nosocomial infections	33 (24.8)
Hemorrhagic events [Type I,II,III]	15 (11.2)[2, 8, 5]
New onset thromboembolic event during ICU stay	17 (12.8)
**Outcomes**	
Ventilatory-free days on day 28	28 [0; 28]
ICU length of stay	7 [4; 14]
Hospital length of stay	15 [8; 23]
ICU mortality	33 (24.8)
Hospital mortality	46 (34.6)
Hospital death and/or invasive mechanical ventilation	59 (44.4)

§Immunosuppression concerned patients with long term or high dosage corticosteroid therapy, anticancer chemotherapy, AIDS, non-AIDS immunodepression, aplasia, bone or organ transplant recipient.

§§ Immunomodulatory treatments comprised IL1 anti receptor, IL6 anti receptor and steroids.

* High-dose prophylactic anticoagulation is defined by higher doses than the standard prophylactic regimen: Enoxaparin 6000 UI/ 24 h if BMI was below 30 kg/ m^2^, enoxaparin 4000 UI/ 12 h if BMI was between 30 and 35 kg/m^2^, and enoxaparin 6000 UI/ 12 h if BMI was above 35 kg/m^2^. Anti-Xa monitoring was performed if clearance was below 20 ml/min and if BMI was above 40 kg/m^2^, in order to avoid overdose.

BMI: Body mass index; HFNC: High flow oxygen nasal cannula, ICU: Intensive care unit; IMV: Invasive mechanical ventilation; IQR: Interquartile range; SAPS: Simplified acute physiology score; SOFA: Sequential organ failure assessment; VFD: Ventilatory free days.

### Laboratory parameters

**Fig 1:**
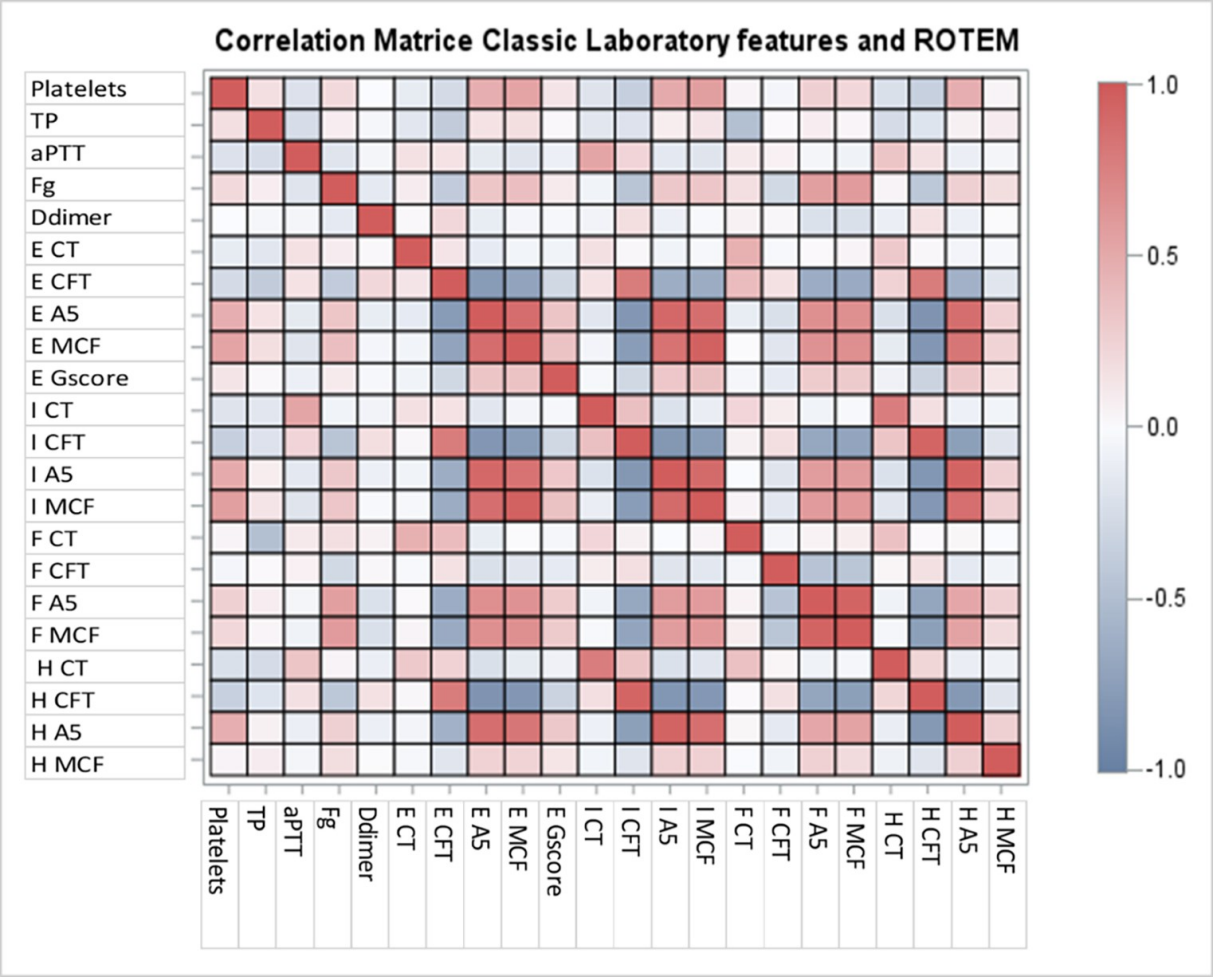
Matrix correlation of the indices from routine coagulation and ROTEM on admission. TP: Prothrombin Time; aPTT: Activated Partial Thromboplastin Time; Fg: Fibrinogen; E: EXTEM; I: INTEM; F: FIBTEM; H: HEPTEM; CT: Clotting time; CFT: Clot formation time; A5: Clot amplitude 5 minutes after CT; MCF: Maximum clot firmness.

The results of blood counts, standard coagulation and ROTEM tests on admission are shown in Table 2. On day 1, the presence of hypercoagulability was observed in 115 (86.4%) patients, with fibrinogen playing a major role in the increased clot firmness. No strong relationship was observed between routine blood coagulation parameters and ROTEM parameters (S1 Table and Fig 1). The analyses between inflammatory parameters and the indices of hypercoagulability are shown in S2 Table. No strong relationship was observed between standard coagulation or ROTEM tests and inflammatory parameters.

**Table 2:** ROTEM, blood count and standard hemostasis on admission.

Laboratory features	*Values*, *med(IQR)*/N(%)
Number of patients	133
**Standard coagulation tests**	
PLATELETS, G/L	254 [190; 315]
PROTHROMBIN TIME (TP), %	84 [75; 92]
ACTIVATED PARTIAL THROMBOPLASTIN TIME (aPTT)	1.2 [1; 1.4]
FIBRINOGEN, g/L	7.2 [6.2; 8]
FIBRINOGEN > 8 g/L	30 (26.6%)
D-DIMERS, μg/dL	1188 [729; 2061.6]
D-DIMERS > 3000 μg/dL	18(16.2%)
**ROTEM variables**	
EXTEM-CT, sec	77 [70; 91]
EXTEM-CT, sec (>80 (Normal range))	62 (46.6)
EXTEM-CFT, sec	48 [42; 56]
EXTEM-CFT, sec (<46 (Normal range))	61 (45.8)
EXTEM-A5, mm	55 [50; 59]
EXTEM-A5, mm (> 52 (Normal range))	89 (67)
EXTEM MCF, mm	73 [69; 75]
EXTEM MCF, mm (> 72 (Normal range))	78 (58.6)
EXTEM Li60, % (missing = 25)	98 [95; 99]
EXTEM Li60, % (>96.5 (Normal range))(miss = 25)	68 (63)
EXTEM G-score§	13.6 [11.2; 15]
EXTEM G-score§ > 11/ G score >14	102 (76.6)/58(43.6)
INTEM CT, sec	186 [167; 206]
FIBTEM MCF, mm	*32 [28; 35]*
HEPTEM CT, sec	184 [170; 201]
Hypercoagulation due to fibrinogenemia§§	131 (98.4)
INTEM CT / HEPTEM CT > 1	69 (51.8)
Presence of at least 1 index in favor of hypercoagulability§§§	115 (86.4)
Presence of at least 4 indices in favor of hypercoagulability	49 (36.8)

§ EXTEM G-score is defined as follows: G-score = (5000xMCF) / (100-MCF).

§§ Hypercoagulation due to fibrinogenemia was defined by a higher difference between observed and normal MCF values of FIBTEM than between observed and normal MCF values of EXTEM.

§§§ Hypercoagulability were defined by at least one of the following indices: Fibrinogen > 8 g/L, D-Dimers > 3000 μg/dL, EXTEM CFT shorter than normal range, EXTEM A5 higher than normal range, EXTEM MCF higher than normal range, G-score ≥11 dyne/cm^2^, and EXTEM Li 60 > 96.5%.

CT: Clotting time; CFT: Clot formation time; A5: Clot amplitude 5 minutes after CT; MCF: Maximum clot firmness; Li60: Lysis index at 60 minutes.

**Fig 2:**
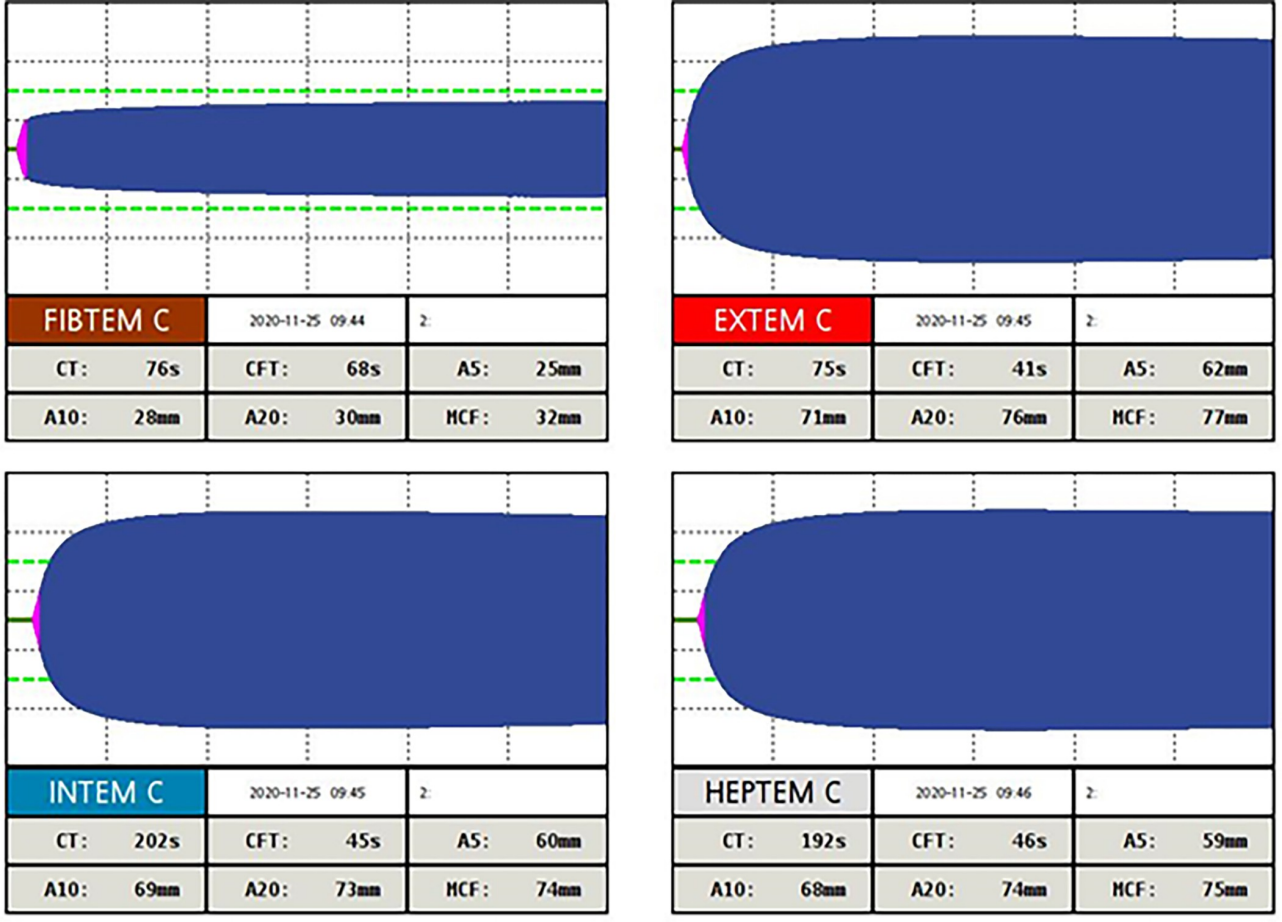
Results of ROTEM tests with the graphical shape performed on admission showing both hypercoagulability and hypofibrinolysis. In this patient, hypercoagulability was characterized by an EXTEM CFT result below normal range values (< 46 s), and EXTEM A5 and EXTEM MCF results above normal range values (> 52 mm and > 72 mm, respectively); hypofibrinolysis was characterized by EXTEM Li 60 > 96.5%. CT: Clotting time; CFT: Clot formation time; A5 –A10 –A20: Clot amplitude 5–10–20 minutes after CT; MCF: Maximum clot firmness.

On day 4, ROTEM was performed in 67 patients. Of these, 66 (98.6%) had ≥ 1 indices of hypercoagulability, including 1 (1.4%) on SPA, 33 (48.6%) on HDPA and 28 (41.2%) on CDA. Of note, hypofibrinolysis was observed in 90.2% of patients. Fig 2 shows the results of ROTEM tests performed on admission in a patient with the graphical shape frequently observed showing both hypercoagulability and hypofibrinolysis.

The results of laboratory tests on day 1 and on day 4 in the 67 patients who underwent both tests are shown in S3 Table. On day 4, as compared to day 1, there was a higher percentage of patients with INTEM CT/HEPTEM CT ratio > 1 (70.2% vs 49.2%, p<0.01), and with ≥1 indices in favor of hypercoagulability (98.6% vs 88%, p = 0.02). Serum fibrinogen values decreased between day 1 and day 4, while all other indices of hypercoagulability had a value on day 4 in favor of a more pronounced state of hypercoagulability than on day 1, as shown by the increase in median values of the platelet count, G-score, EXTEM A5, EXTEM MCF, EXTEM Li60, and the decrease in median values of EXTEM CFT (S3 Table).

### Factors associated with TE events

**Fig 3:**
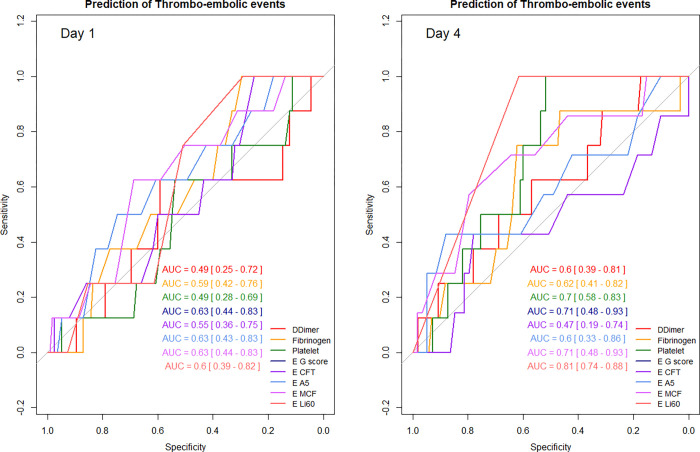
Prediction of the occurrence of thrombo-embolic events during ICU stay by coagulation indices on day 1 and day 4. AUC: Area Under the Curve; E: EXTEM; CFT: Clot formation time; A5: Clot amplitude 5 minutes after CT; MCF: Maximum clot firmness; Li60: Lysis index at 60 minutes.

None of the indices of hypercoagulability measured on day 1 or on day 4 were associated with new- onset TE events. Conversely, higher serum IL-6 values were (S4 Table). The ability of D-dimers, fibrinogen, platelet count, EXTEM CFT, A5, MCF, G-score, and Li 60 to predict the occurrence of new-onset TE events was assessed on days 1 and 4. All these parameters had a very low performance on day 1 (all AUC ROC curves < 0.7). On day 4, EXTEM Li 60 (AUC = 0.81 [0.74–0.88]), EXTEM MCF (AUC = 0.71 [0.48–0.93]), EXTEM G-score (AUC = 0.71 [0.48–0.93]) and platelet (AUC = 0.7 [0.58–0.83]) were the parameters associated with a greater ability to predict the occurrence of TE events (Fig 3, S5 Table).

### Factors associated with intubation

**Fig 4:**
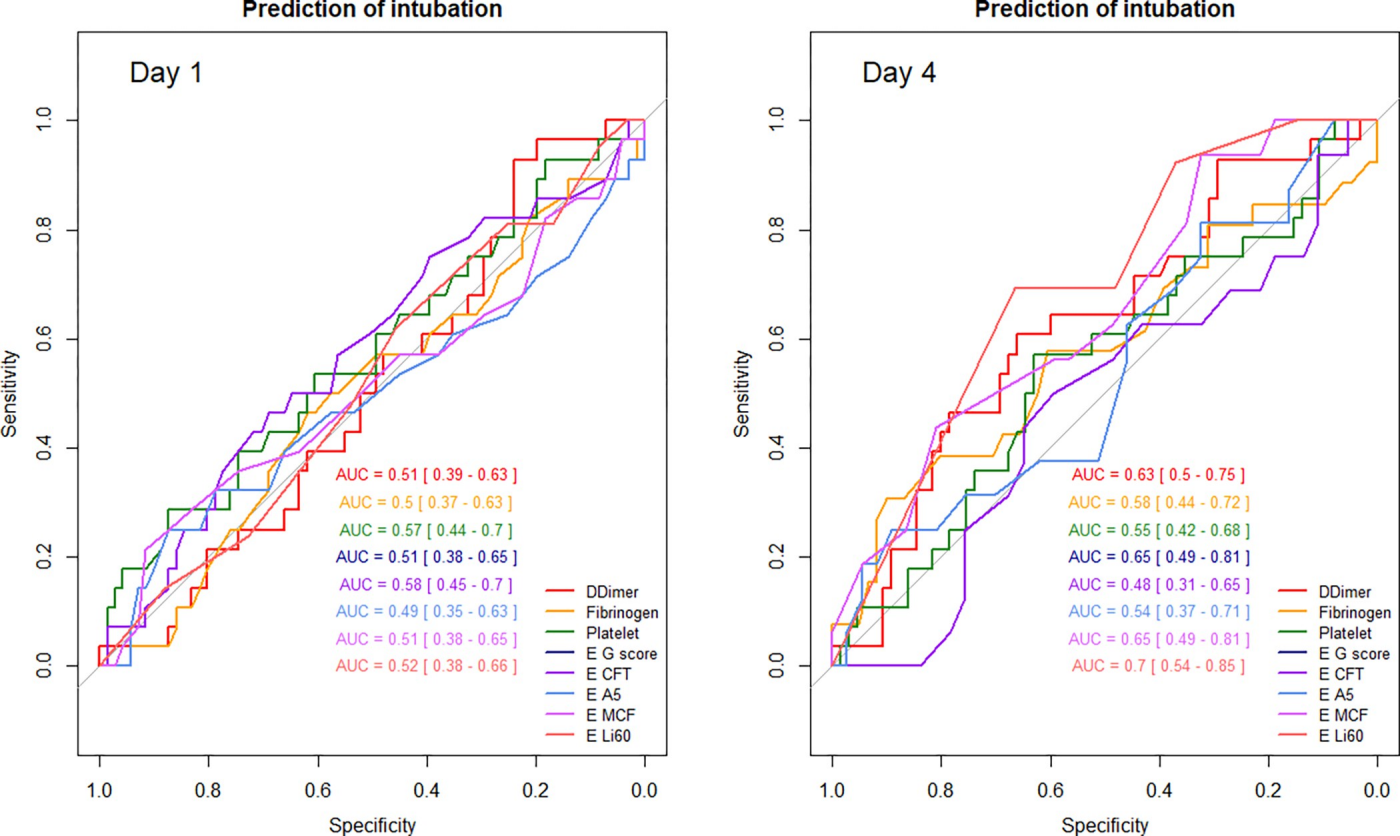
Prediction of intubation during ICU stay by coagulation indices on day 1 and day 4. AUC: Area Under the Curve; E: EXTEM; CFT: Clot formation time; A5: Clot amplitude 5 minutes after clotting time; MCF: Maximum clot firmness; Li60: Lysis index at 60 minutes.

The factors associated on day 1 with an increased risk of intubation were only higher serum IL-6 and Il-10 values and lower mHLA-DR. On day 4, high fibrinogen (p = 0.02) and high Li60 (p = 0.05) were associated with intubation during ICU stay. All the indices measured on days 1 or 4 had a very low ability (all AUC ROC curves < 0.7) to predict the need for intubation except for Li60 on day 4 (Fig 4, S6 and S7 Tables).

### Factors associated with ICU death

**Fig 5:**
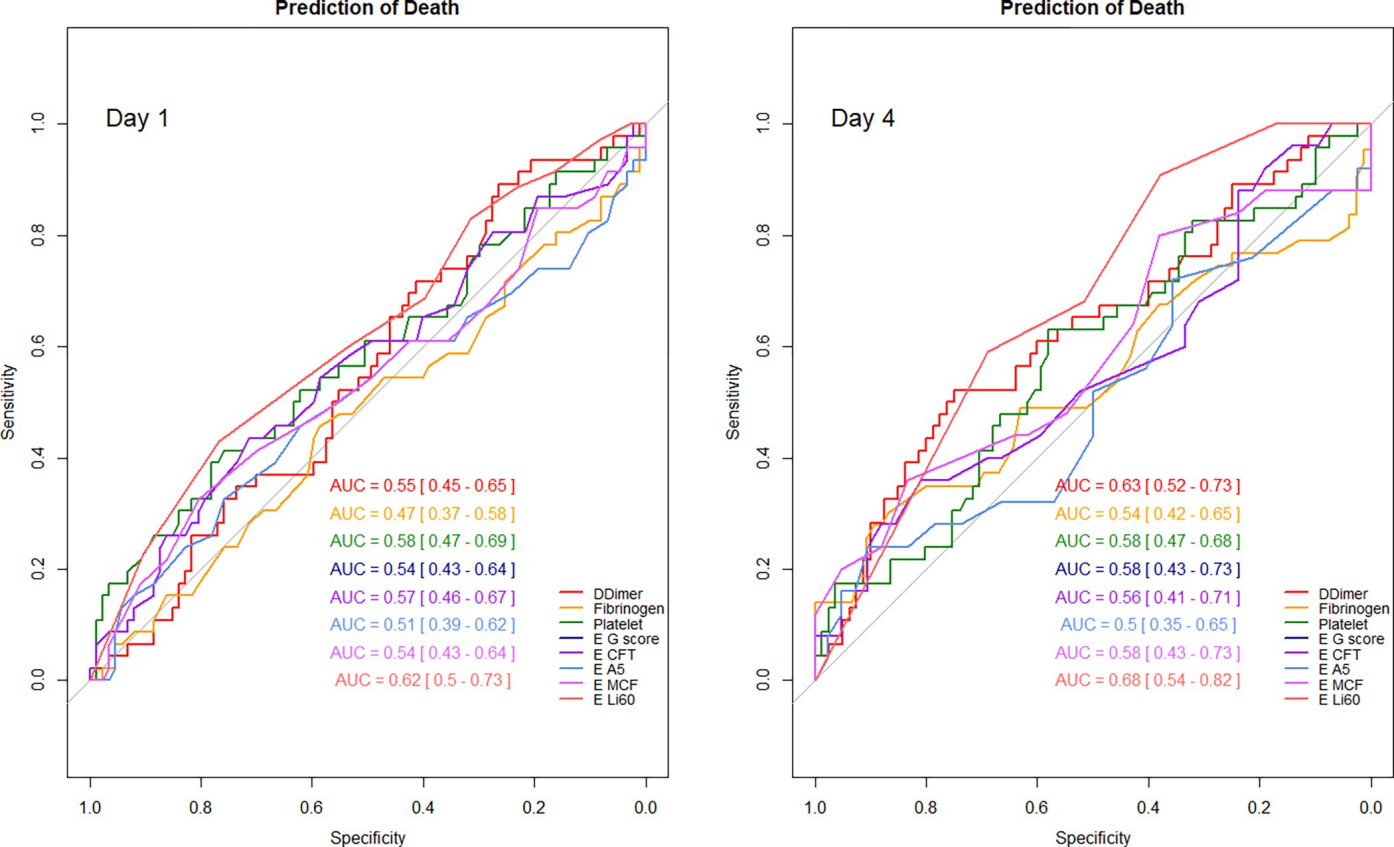
Prediction of ICU death by coagulation indices on day 1 and day 4. AUC: Area Under the Curve; E: EXTEM; CFT: Clot formation time; A5: Clot amplitude 5 minutes after clotting time; MCF: Maximum clot firmness; Li60: Lysis index at 60 minutes.

The factors associated on day 1 with an increased risk of ICU death was EXTEM Li60 (p = 0.05), and on day 4 higher were serum values of fibrinogen (p = 0.02), D-dimers (p = 0.04), and EXTEM Li 60 (p = 0.03). On day 1, higher serum IL-6 and Il-10 values and lower mHLA-DR values were associated with ICU death (S8 Table). All the indices measured on day 1 or 4 had a very low ability (all AUC ROC curves < 0.7) to predict the occurrence of ICU death (Fig 5, S9 Table).

### Factors associated with intubation and/or ICU death

**Fig 6:**
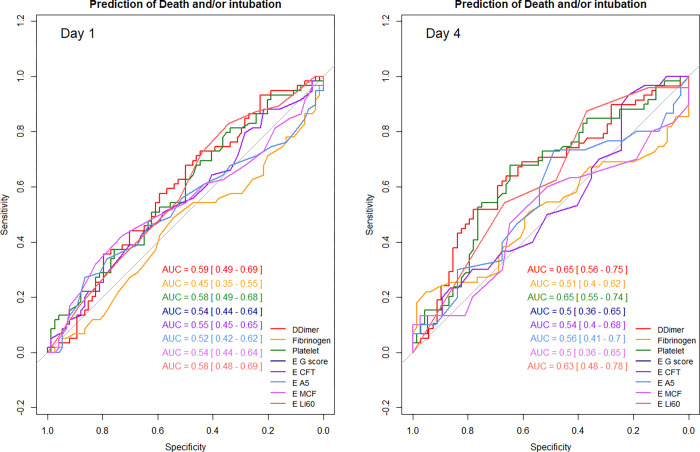
Prediction of intubation and/or ICU death by coagulation indices on day 1 and day 4. AUC: Area Under the Curve; E: EXTEM; CFT: Clot formation time; A5: Clot amplitude 5 minutes after clotting time; MCF: Maximum clot firmness; Li60: Lysis index at 60 minutes.

The factors associated with an increased risk of intubation and/or ICU death on day 1 were INTEM CT/ HEPTEM CT ratio > 1 (p = 0.03), and on day 4 higher serum values of fibrinogen (p = 0.02), of D-dimers (p = 0.04), and of EXTEM Li 60 (p = 0.03). On day 1, higher serum IL-10 values and lower mHLA-DR values were associated with death or intubation (S10 Table). All the indices measured on day 1 or on day 4 had a very low ability (all AUC ROC curves < 0.7) to predict the occurrence of death and/or intubation (Fig 6, S10 and S11 Tables).

### Bleeding

Major bleeding complications were observed in 15 (11.30%) patients during ICU stay (Table 1). No bleeding led to death.

## Discussion

In this cohort, designed to characterize hypercoagulability in COVID-19 ICU patients using routine blood coagulation and ROTEM parameters, 21% of patients experienced a new TE event, 31% were intubated and 35% died in hospital. We confirmed hypercoagulability in COVID with fibrinogen playing a predominant role. ROTEM indices were not strongly correlated with results of standard blood tests. EXTEM Li 60, EXTEM MCF, EXTEM G-score and platelet were the most reliable indices to predict the occurrence of TE events, whereas hyperfibrinogenemia, higher D-dimer and higher EXTEM Li 60 levels on day 4 were associated with an increased risk of death and intubation. Finally, anticoagulation on admission as evidenced by an INTEM-CT/ HEPTEM-CT >1 was associated with an increased risk of death or intubation.

We observed a lower rate of TE events in earlier studies, which occurred in 30 to 35% of COVID-19 ICU patients and were mainly cases of pulmonary embolism [39–41]. These figures were confirmed in a recent meta-analysis of 15 studies that recorded a 27.9% rate of venous TE events among COVID-19 ICU patients [42]. However, a recent large study also found lower rates of TE events, of 6.4% and 10.4%, in patients with preventive and curative anticoagulation, respectively [43]. This lower rate could be explained by the study period: most patients were enrolled after July 2020 and were prescribed high-dose prophylactic or curative anticoagulation, as then recommended [5,15,17,44–46], and also steroids, which could have minimized inflammation and associated hypercoagulation [47]. In addition, since follow-up CT scans and Doppler ultrasound were only performed in our ICU when suspected from the evolution of clinical or laboratory parameters, we cannot exclude that we underdiagnosed the occurrence of a new TE event. In our study, like most authors, we found no strong correlations between standard blood tests and ROTEM [48–50].

ROTEM showed biological hypercoagulability in our COVID-19 ICU patients on days 1 and 4, which combined increased clot propagation and firmness, and hypofibrinolysis. Similar results have been previously reported in the ICU [13,35,51–55]. For instance, Saseedharan et al. [56] and Yuriditsky et al.[51] retrospectively observed hypercoagulation based on the thromboelastography profile of 62.5% and 50% among their cohorts of 32 and 64 COVID-19 ICU patients, respectively. Almskog et al. found a hypercoagulation thromboelastometry profile with EXTEM-MCF and FIBTEM-MCF significantly higher in 60 hospitalized COVID-19 patients (including 20 ICU patients) than in healthy volunteers. A higher state of hypercoagulability on ROTEM profiles was also reported in COVID patients admitted to the ICU than in less severely ill patients admitted to medical wards [35,57].

In our study we observed that hypercoagulation was mostly due to both hyperfibrinogenemia, and hypofibrinolysis. Although not systematically reported by authors [13,58], fibrinolysis shutdown has been documented as playing a predominant role in COVID-19 hypercoagulability [59–63], driven either by COVID or by the severity of the patient’s condition [64].

In our study, among the hypercoagulability indices, hypofibrinolysis on day 4 as defined by elevated Li60 values had the best accuracy to predict new-onset TE events. The association between fibrinolysis shutdown and risk of TE events in COVID-19 ICU patients has been previously reported. In a prospective study of 44 ICU patients, Wright et al. described an association between thromboelastography fibrinolysis shutdown defined by a zero value of the Li30 parameter and venous TE events (AUC 0.742). In addition, they proposed a combined score of Li30 values and D-dimer levels to estimate the risk of venous TE events during ICU stay [59]. Similarly, in a prospective study of 40 COVID-19 ICU patients, Kruse et al. reported an exacerbated venous TE risk associated with low EXTEM and INTEM maximum lysis (ML) values, with AUCs for both tests of 0.8. The authors also reported that the difference between EXTEM ML and maximum D-dimer levels predicted TE risk with an AUC of 0.9 [63]. Taken together these findings support a predominant role of reduced fibrinolysis in the development of venous TE events in severe COVID-19. In our study, serum fibrinogen and D-dimer levels, in contrast to Li60 values, failed to predict TE events, underlining the better sensitivity of ROTEM indices over standard blood tests in this setting.

On day 4, hyperfibrinogenemia, and elevated D-dimer levels and Li60 values, which can all reflect greater fibrinolysis resistance, were associated with an increased risk of intubation and/or ICU death. In COVID ICU patients, the association between unfavorable outcome and elevated fibrinogen [65] and D-dimer levels [66–68] has been previously reported. Similarly, Roh et al. suggested that the significant increase in fibrinogen plasma concentrations and FIBTEM MCF indicates the severity of COVID-19 and can be used for risk stratification for thrombosis, respiratory failure, and mortality [66]. The relationship between Li60 values and mortality remains debatable [32]: it has been reported in septic patients, but never to our knowledge in COVID-19 ICU patients [36].

In our study, we observed prolonged clot initiation. Whether it can be related to heparin exposure requires a careful analysis since the impact of heparin on INTEM-CT depends on both the type of heparin and its dosage. While ROTEM tests are able to detect the presence of UFH on the basis of an increase in the difference, or in the ratio, between INTEM-CT and HEPTEM-CT, and whereas in patients administered with UFH a prolonged INTEM-CT is reported for heparinemia > 0.1 U/l, both traditional coagulative parameters and ROTEM profiles are poorly influenced by LMWH, and an increased INTEM-CT in patients receiving LMWH is only observed for heparinemia >0.4UI/l [69,70]. We observed similar results in our study. INTEM/HEPTEM CT > 1 was detected in patients receiving UFH for HDPA or CDA, and in 90% of patients under LMWH for CDA, but only in half of the patients under LMWH for HDPA (S12 Table).

Despite HDPA and even CDA, hypercoagulability was more pronounced on day 4 than on day 1. These results are in close agreement with those of previous studies [32,71]. Our data support the results of REMAP-CAP demonstrating the absence of benefit of CDA in COVID ICU patients [43]. One possible explanation is that CDA was given too late to reverse the consequences of the disease process in severe COVID-19 patients. In line with these results, we also observed that an INTEM-CT longer than an HEPTEM-CT, which was mostly observed in patients administered with HDPA or CDA UFH, or with CDA LMWH, was associated with an increased risk of intubation and/or ICU death. In our cohort, CDA was administered to patients with documented TE events and in those with elevated D-dimer and/or fibrinogen levels. We cannot therefore exclude that these criteria identify the most severely ill patients. Furthermore, in our cohort, most of the time, the patients under UFH are the most severely ill patients, i.e., those receiving mechanical ventilation or renal replacement therapy. This could also contribute to the relationship between the ratio INTEM/HEPTEM CT > 1 and worse outcomes in our cohort.

However, even though major bleeding events were few and unrelated to CDA, it is possible that in some patients CDA had been harmful by exacerbating alveolar hemorrhage.

The main strength of this study is to be one of the largest cohorts of critically ill COVID patients monitored by ROTEM on admission and on day 4. However, it also has several limitations. First, it was a monocentric study involving a cohort of patients admitted during the second and third waves in France, and therefore most of the patients were on steroids and receiving protocolized anticoagulation, which limits comparisons with the first published studies. Second, ROTEM was performed on day 4 in only 60% of our patients, mostly because of death or living alive patients. Third, we chose day 4 as the onset of inflammation whereas other authors proposed day 7 [37]. Finally, several TE events might have been missed because of the retrospective recording of their occurrence.

## Conclusions

Our study confirmed that a majority of critically ill COVID-19 patients had hypercoagulability as a result of hyperfibrinogenemia mostly secondary to hypofibrinolysis. Routine blood coagulation and ROTEM parameters were poorly correlated. On day 4, the accuracy of Li60 to predict new onset TE events was good, and hyperfibrinogenemia and Li60 values were good indices to identify the patients the most at risk of intubation and/or ICU death. Our findings question the systematic curative anticoagulation strategy proposed in COVID-19 patients with elevated fibrinogen and/or D-dimer levels.

## Supporting information

S1 Table Coefficient correlation matrix between indices from ROTEM and others from usual laboratory features. TP: Prothrombin Time; aPTT: Activated Partial Thromboplastin Time; Fg: Fibrinogen; E: EXTEM; I: INTEM; F: FIBTEM; H: HEPTEM; CT: Clotting time; CFT: Clot formation time; A5: Clot amplitude at 5 minutes; MCF: Maximum clot firmness.
(DOCX)

S2 Table Coefficient correlation matrix between indices of hypercoagulability and inflammatory parameters. CRP: C reactive protein; Fg: Fibrinogen; E: EXTEM; CFT: Clot formation time; A5: Clot amplitude at 5 minutes; MCF: Maximum clot firmness; Li60: Lysis index at 60 minutes.
(DOCX)

S3 Table Comparison between day 1 and day 4 for standard hemostasis and ROTEM. § EXTEM G-score is defined as follows: G-score = (5000xMCF) / (100-MCF). §§ Hypercoagulation due to fibrinogenemia was defined by a higher difference between observed and normal MCF values of FIBTEM than between observed and normal MCF values of EXTEM. §§§ Hypercoagulability were defined by at least one of the following indices: Fibrinogen > 8 g/L, D-Dimers > 3000 μg/dL, EXTEM CFT shorter than normal range, EXTEM A5 higher than normal range, EXTEM MCF higher than normal range, G score ≥11 dyne/cm2, and EXTEM Li 60 > 96.5%. CFT: Clot formation time; A5: Clot amplitude at 5 minutes; MCF: Maximum clot firmness; Li60: Lysis index at 60 minutes.
(DOCX)

S4 Table Factors associated with the occurrence of thrombo-embolic events during intensive care stay. TE: Thrombo-embolic event; CFT: Clot formation time; A5: Clot amplitude at 5 minutes; MCF: Maximum clot firmness; Li60: Lysis index at 6 minutes; CRP: C reactive protein.
(DOCX)

S5 Table Prediction of occurrence of thrombo-embolic events during ICU stay by coagulation indices on days 1 and 4. AUC: Area under the curve; CFT: Clot formation time; A5: Clot amplitude at 5 minutes; MCF: Maximum clot firmness; Li60: Lysis index at 60 minutes.
(DOCX)

S6 Table Factors associated with intubation during ICU stay among patients non intubated on admission. CFT: Clot formation time; A5: Clot amplitude at 5 minutes; MCF: Maximum clot firmness; Li60: Lysis index at 60 minutes.
(DOCX)

S7 Table Prediction of intubation during ICU stay by coagulation indices on days 1 and 4. AUC: Area under the curve; CFT clot formation time; A5: Clot amplitude at 5 minutes; MCF: Maximum clot formation time; Li60: Lysis index at 60 minutes.
(DOCX)

S8 Table Comparison of patients who experienced ICU death and those who did not. CFT: Clot formation time; A5: Clot amplitude at 5 minutes; MCF: Maximum clot firmness; Li60: Lysis index at 60 minutes.
(DOCX)

S9 Table Prediction of occurrence of death by coagulation indices on days 1 and 4. AUC: Area under the curve; CFT clot formation time; A5: Clot amplitude at 5 minutes; MCF: Maximum clot formation time; Li60: Lysis index at 60 minutes.
(DOCX)

S10 Table Comparison of patients who experienced hospital death and/or invasive mechanical ventilation and those who did not. CFT: Clot formation time; A5: Clot amplitude at 5 minutes; MCF: Maximum clot firmness; Li60: Lysis index at 60 minutes.
(DOCX)

S11 Table Prediction of occurrence of death and/or intubation by coagulation indices on days 1 and 4. AUC: Area under the curve; CFT clot formation time; A5: Clot amplitude at 5 minutes; MCF: Maximum clot formation time; Li60: Lysis index at 60 minutes.
(DOCX)

S12 Table Anticoagulation and prolonged CT on day 4. HDPA: High dose preventive anticoagulation; CDA: Curative dose anticoagulation.
(DOCX)

S1 File Supplemental file–definitions. (DOCX)

S1 Data (XLSX)

## References

[1] Piazza G, Morrow DA. Diagnosis, Management, and Pathophysiology of Arterial and Venous Thrombosis in COVID-19. JAMA. 2020;324: 2548–2549. doi: 10.1001/jama.2020.23422 33226423

[2] Chen N, Zhou M, Dong X, Qu J, Gong F, Han Y, et al. Epidemiological and clinical characteristics of 99 cases of 2019 novel coronavirus pneumonia in Wuhan, China: a descriptive study. Lancet. 2020;395: 507–513. doi: 10.1016/S0140-6736(20)30211-7 32007143 PMC7135076

[3] Tang N, Bai H, Chen X, Gong J, Li D, Sun Z. Anticoagulant treatment is associated with decreased mortality in severe coronavirus disease 2019 patients with coagulopathy. J Thromb Haemost. 2020;18: 1094–1099. doi: 10.1111/jth.14817 32220112 PMC9906401

[4] Danzi GB, Loffi M, Galeazzi G, Gherbesi E. Acute pulmonary embolism and COVID-19 pneumonia: a random association? Eur Heart J. 2020;41: 1858. doi: 10.1093/eurheartj/ehaa254 32227120 PMC7184406

[5] Bikdeli B, Madhavan MV, Jimenez D, Chuich T, Dreyfus I, Driggin E, et al. COVID-19 and Thrombotic or Thromboembolic Disease: Implications for Prevention, Antithrombotic Therapy, and Follow-Up: JACC State-of-the-Art Review. J Am Coll Cardiol. 2020;75: 2950–2973. doi: 10.1016/j.jacc.2020.04.031 32311448 PMC7164881

[6] Guo T, Fan Y, Chen M, Wu X, Zhang L, He T, et al. Cardiovascular Implications of Fatal Outcomes of Patients With Coronavirus Disease 2019 (COVID-19). JAMA Cardiol. 2020;5: 811–818. doi: 10.1001/jamacardio.2020.1017 32219356 PMC7101506

[7] Ackermann M, Verleden SE, Kuehnel M, Haverich A, Welte T, Laenger F, et al. Pulmonary Vascular Endothelialitis, Thrombosis, and Angiogenesis in Covid-19. New England Journal of Medicine. 2020;383: 120–128. doi: 10.1056/NEJMoa2015432 32437596 PMC7412750

[8] Driggin E, Madhavan MV, Bikdeli B, Chuich T, Laracy J, Biondi-Zoccai G, et al. Cardiovascular Considerations for Patients, Health Care Workers, and Health Systems During the COVID-19 Pandemic. J Am Coll Cardiol. 2020;75: 2352–2371. doi: 10.1016/j.jacc.2020.03.031 32201335 PMC7198856

[9] Ackermann M, Verleden SE, Kuehnel M, Haverich A, Welte T, Laenger F, et al. Pulmonary Vascular Endothelialitis, Thrombosis, and Angiogenesis in Covid-19. N Engl J Med. 2020;383: 120–128. doi: 10.1056/NEJMoa2015432 32437596 PMC7412750

[10] Helms J, Tacquard C, Severac F, Leonard-Lorant I, Ohana M, Delabranche X, et al. High risk of thrombosis in patients with severe SARS-CoV-2 infection: a multicenter prospective cohort study. Intensive Care Med. 2020;46: 1089–1098. doi: 10.1007/s00134-020-06062-x 32367170 PMC7197634

[11] Klok FA, Kruip MJHA, van der Meer NJM, Arbous MS, Gommers D MPJ a, Kant KM, et al. Incidence of thrombotic complications in critically ill ICU patients with COVID-19. Thromb Res. 2020;191: 145–147. doi: 10.1016/j.thromres.2020.04.013 32291094 PMC7146714

[12] van Veenendaal N, Scheeren TWL, Meijer K, van der Voort PHJ. Rotational thromboelastometry to assess hypercoagulability in COVID-19 patients. Thromb Res. 2020;196: 379–381. doi: 10.1016/j.thromres.2020.08.046 32980621 PMC7462575

[13] Pavoni V, Gianesello L, Pazzi M, Stera C, Meconi T, Frigieri FC. Evaluation of coagulation function by rotation thromboelastometry in critically ill patients with severe COVID-19 pneumonia. J Thromb Thrombolysis. 2020;50: 281–286. doi: 10.1007/s11239-020-02130-7 32394236 PMC7211560

[14] Mondal S, Quintili AL, Karamchandani K, Bose S. Thromboembolic disease in COVID-19 patients: A brief narrative review. J Intensive Care. 2020;8: 70. doi: 10.1186/s40560-020-00483-y 32939266 PMC7487447

[15] Susen S, Tacquard CA, Godon A, Mansour A, Garrigue D, Nguyen P, et al. Prevention of thrombotic risk in hospitalized patients with COVID-19 and hemostasis monitoring. Crit Care. 2020;24: 364. doi: 10.1186/s13054-020-03000-7 32560658 PMC7303590

[16] Gerotziafas GT, Catalano M, Colgan M-P, Pecsvarady Z, Wautrecht JC, Fazeli B, et al. Guidance for the Management of Patients with Vascular Disease or Cardiovascular Risk Factors and COVID-19: Position Paper from VAS-European Independent Foundation in Angiology/Vascular Medicine. Thromb Haemost. 2020;120: 1597–1628. doi: 10.1055/s-0040-1715798 32920811 PMC7869052

[17] Moores LK, Tritschler T, Brosnahan S, Carrier M, Collen JF, Doerschug K, et al. Prevention, Diagnosis, and Treatment of VTE in Patients With Coronavirus Disease 2019: CHEST Guideline and Expert Panel Report. Chest. 2020;158: 1143–1163. doi: 10.1016/j.chest.2020.05.559 32502594 PMC7265858

[18] Thomas W, Samama C-M, Greinacher A, Hunt BJ, Subcommittee on Perioperative and Critical Care. The utility of viscoelastic methods in the prevention and treatment of bleeding and hospital-associated venous thromboembolism in perioperative care: guidance from the SSC of the ISTH. J Thromb Haemost. 2018;16: 2336–2340. doi: 10.1111/jth.14265 30171663

[19] Spahn DR, Bouillon B, Cerny V, Duranteau J, Filipescu D, Hunt BJ, et al. The European guideline on management of major bleeding and coagulopathy following trauma: fifth edition. Crit Care. 2019;23. doi: 10.1186/s13054-019-2347-3 30917843 PMC6436241

[20] Collins P, Abdul-Kadir R, Thachil J, Subcommittees on Women’ s Health Issues in Thrombosis and Haemostasis and on Disseminated Intravascular Coagulation. Management of coagulopathy associated with postpartum hemorrhage: guidance from the SSC of the ISTH. J Thromb Haemost. 2016;14: 205–210. doi: 10.1111/jth.13174 27028301

[21] Taura P, Rivas E, Martinez-Palli G, Blasi A, Holguera JC, Balust J, et al. Clinical markers of the hypercoagulable state by rotational thrombelastometry in obese patients submitted to bariatric surgery. Surg Endosc. 2014;28: 543–551. doi: 10.1007/s00464-013-3203-1 24043645

[22] Gurunathan U, Stanton LM, Weir RM, Hay KE, Pearse BL. A preliminary study using rotational thromboelastometry to investigate perioperative coagulation changes and to identify hypercoagulability in obese patients undergoing total hip or knee replacement. Anaesth Intensive Care. 2019;47: 461–468. doi: 10.1177/0310057X19864114 31537080

[23] Baranich AI, Polupan AA, Sychev AA, Savin IA, Tabasaranskiy TF, Kurdumova NV, et al. Thromboelastometry as a Comprehensive Assessment of Hypercoagulation After Aneurysmal Subarachnoid Hemorrhage: A Case Report and Literature Review. Acta Neurochir Suppl. 2020;127: 165–169. doi: 10.1007/978-3-030-04615-6_25 31407079

[24] Thorson CM, Van Haren RM, Ryan ML, Curia E, Sleeman D, Levi JU, et al. Pre-existing hypercoagulability in patients undergoing potentially curative cancer resection. Surgery. 2014;155: 134–144. doi: 10.1016/j.surg.2013.06.053 24238121

[25] Dimitrova-Karamfilova A. Rotation Thromboelastography for Assessment of Hypercoagulation and Thrombosis in Patients with Cardiovascular Diseases. J Life Sci. 2012; 28–35.

[26] Hincker A, Feit J, Sladen RN, Wagener G. Rotational thromboelastometry predicts thromboembolic complications after major non-cardiac surgery. Crit Care. 2014;18: 549. doi: 10.1186/s13054-014-0549-2 25292221 PMC4200117

[27] Zanetto A, Senzolo M, Vitale A, Cillo U, Radu C, Sartorello F, et al. Thromboelastometry hypercoagulable profiles and portal vein thrombosis in cirrhotic patients with hepatocellular carcinoma. Dig Liver Dis. 2017;49: 440–445. doi: 10.1016/j.dld.2016.12.019 28109767

[28] Kamel Y, Hassanin A, Ahmed AR, Gad E, Afifi M, Khalil M, et al. Perioperative Thromboelastometry for Adult Living Donor Liver Transplant Recipients with a Tendency to Hypercoagulability: A Prospective Observational Cohort Study. Transfus Med Hemother. 2018;45: 404–412. doi: 10.1159/000489605 30574058 PMC6288633

[29] Schaden E, Schober A, Hacker S, Spiss C, Chiari A, Kozek-Langenecker S. Determination of enoxaparin with rotational thrombelastometry using the prothrombinase-induced clotting time reagent. Blood Coagul Fibrinolysis. 2010;21: 256–261. doi: 10.1097/MBC.0b013e328337014c 20087172

[30] Corrêa TD, Cordioli RL, Campos Guerra JC, Caldin da Silva B, Dos Reis Rodrigues R, de Souza GM, et al. Coagulation profile of COVID-19 patients admitted to the ICU: An exploratory study. PLoS One. 2020;15: e0243604. doi: 10.1371/journal.pone.0243604 33320874 PMC7737963

[31] Heinz C, Miesbach W, Herrmann E, Sonntagbauer M, Raimann FJ, Zacharowski K, et al. Greater Fibrinolysis Resistance but No Greater Platelet Aggregation in Critically Ill COVID-19 Patients. Anesthesiology. 2021;134: 457–467. doi: 10.1097/ALN.0000000000003685 33417674 PMC7864605

[32] Hvas CL, Larsen JB, Adelborg K, Christensen S, Hvas A-M. Dynamic Hemostasis and Fibrinolysis Assays in Intensive Care COVID-19 Patients and Association with Thrombosis and Bleeding-A Systematic Review and a Cohort Study. Semin Thromb Hemost. 2022;48: 31–54. doi: 10.1055/s-0041-1735454 34715692

[33] Creel-Bulos C, Auld SC, Caridi-Scheible M, Barker NA, Friend S, Gaddh M, et al. Fibrinolysis Shutdown and Thrombosis in a COVID-19 ICU. Shock. 2021;55: 316–320. doi: 10.1097/SHK.0000000000001635 32769822 PMC8858425

[34] Almskog LM, Wikman A, Svensson J, Wanecek M, Bottai M, van der Linden J, et al. Rotational thromboelastometry results are associated with care level in COVID-19. J Thromb Thrombolysis. 2021;51: 437–445. doi: 10.1007/s11239-020-02312-3 33068277 PMC7568025

[35] Almskog LM, Wikman A, Svensson J, Wanecek M, Bottai M, van der Linden J, et al. Rotational thromboelastometry results are associated with care level in COVID-19. J Thromb Thrombolysis. 2021;51: 437–445. doi: 10.1007/s11239-020-02312-3 33068277 PMC7568025

[36] Snegovskikh D, Kendall MC, Levinson A, Sarpatwari R, Pisano D, Görlinger K, et al. Thromboelastometry Predicts Thromboembolic Events, Hospital Length of Stay, and Mortality in Patients with COVID-19 Infection and Mild Hypoxemia: A Prospective Observational Study. J Blood Med. 2022;13: 363–372. doi: 10.2147/JBM.S363999 35789896 PMC9250315

[37] Bonnet B, Cosme J, Dupuis C, Coupez E, Adda M, Calvet L, et al. Severe COVID-19 is characterized by the co-occurrence of moderate cytokine inflammation and severe monocyte dysregulation. EBioMedicine. 2021;73: 103622. doi: 10.1016/j.ebiom.2021.103622 34678611 PMC8526358

[38] Schulman S, Kearon C, Subcommittee on Control of Anticoagulation of the Scientific and Standardization Committee of the International Society on Thrombosis and Haemostasis. Definition of major bleeding in clinical investigations of antihemostatic medicinal products in non-surgical patients. J Thromb Haemost. 2005;3: 692–694. doi: 10.1111/j.1538-7836.2005.01204.x 15842354

[39] Piazza G, Campia U, Hurwitz S, Snyder JE, Rizzo SM, Pfeferman MB, et al. Registry of Arterial and Venous Thromboembolic Complications in Patients With COVID-19. J Am Coll Cardiol. 2020;76: 2060–2072. doi: 10.1016/j.jacc.2020.08.070 33121712 PMC7588178

[40] Klok FA, Kruip MJHA, van der Meer NJM, Arbous MS, Gommers D, Kant KM, et al. Confirmation of the high cumulative incidence of thrombotic complications in critically ill ICU patients with COVID-19: An updated analysis. Thromb Res. 2020;191: 148–150. doi: 10.1016/j.thromres.2020.04.041 32381264 PMC7192101

[41] Léonard-Lorant I, Delabranche X, Séverac F, Helms J, Pauzet C, Collange O, et al. Acute Pulmonary Embolism in Patients with COVID-19 at CT Angiography and Relationship to d-Dimer Levels. Radiology. 2020;296: E189–E191. doi: 10.1148/radiol.2020201561 32324102 PMC7233397

[42] Jiménez D, García-Sanchez A, Rali P, Muriel A, Bikdeli B, Ruiz-Artacho P, et al. Incidence of VTE and Bleeding Among Hospitalized Patients With Coronavirus Disease 2019. Chest. 2021;159: 1182–1196. doi: 10.1016/j.chest.2020.11.005 33217420 PMC7670889

[43] REMAP-CAP Investigators, ACTIV-4a Investigators, ATTACC Investigators, Goligher EC, Bradbury CA, McVerry BJ, et al. Therapeutic Anticoagulation with Heparin in Critically Ill Patients with Covid-19. N Engl J Med. 2021;385: 777–789. doi: 10.1056/NEJMoa2103417 34351722 PMC8362592

[44] Zhai Z, Li C, Chen Y, Gerotziafas G, Zhang Z, Wan J, et al. Prevention and Treatment of Venous Thromboembolism Associated with Coronavirus Disease 2019 Infection: A Consensus Statement before Guidelines. Thromb Haemost. 2020;120: 937–948. doi: 10.1055/s-0040-1710019 32316065 PMC7295267

[45] Thachil J, Agarwal S. Understanding the COVID-19 coagulopathy spectrum. Anaesthesia. 2020;75: 1432–1436. doi: 10.1111/anae.15141 32437586 PMC7280700

[46] Flaczyk A, Rosovsky RP, Reed CT, Bankhead-Kendall BK, Bittner EA, Chang MG. Comparison of published guidelines for management of coagulopathy and thrombosis in critically ill patients with COVID 19: implications for clinical practice and future investigations. Crit Care. 2020;24: 559. doi: 10.1186/s13054-020-03273-y 32938471 PMC7492793

[47] RECOVERY Collaborative Group, Horby P, Lim WS, Emberson JR, Mafham M, Bell JL, et al. Dexamethasone in Hospitalized Patients with Covid-19. N Engl J Med. 2021;384: 693–704. doi: 10.1056/NEJMoa2021436 32678530 PMC7383595

[48] Masi P, Hékimian G, Lejeune M, Chommeloux J, Desnos C, Pineton De Chambrun M, et al. Systemic Inflammatory Response Syndrome Is a Major Contributor to COVID-19-Associated Coagulopathy: Insights From a Prospective, Single-Center Cohort Study. Circulation. 2020;142: 611–614. doi: 10.1161/CIRCULATIONAHA.120.048925 32776849 PMC7418760

[49] Kamel Y, Hassanin A, Ahmed AR, Gad E, Afifi M, Khalil M, et al. Perioperative Thromboelastometry for Adult Living Donor Liver Transplant Recipients with a Tendency to Hypercoagulability: A Prospective Observational Cohort Study. Transfus Med Hemother. 2018;45: 404–412. doi: 10.1159/000489605 30574058 PMC6288633

[50] Roh DJ, Eiseman K, Kirsch H, Yoh N, Boehme A, Agarwal S, et al. Hypercoagulable viscoelastic blood clot characteristics in critically ill coronavirus disease 2019 patients and associations with thrombotic complications. J Trauma Acute Care Surg. 2021;90: e7–e12. doi: 10.1097/TA.0000000000002963 33009340 PMC8630999

[51] Yuriditsky E, Horowitz JM, Merchan C, Ahuja T, Brosnahan SB, McVoy L, et al. Thromboelastography Profiles of Critically Ill Patients With Coronavirus Disease 2019. Crit Care Med. 2020;48: 1319–1326. doi: 10.1097/CCM.0000000000004471 32618696 PMC7314320

[52] Ranucci M, Ballotta A, Di Dedda U, Baryshnikova E, Dei Poli M, Resta M, et al. The procoagulant pattern of patients with COVID-19 acute respiratory distress syndrome. J Thromb Haemost. 2020;18: 1747–1751. doi: 10.1111/jth.14854 32302448 PMC9906332

[53] Mortus JR, Manek SE, Brubaker LS, Loor M, Cruz MA, Trautner BW, et al. Thromboelastographic Results and Hypercoagulability Syndrome in Patients With Coronavirus Disease 2019 Who Are Critically Ill. JAMA Netw Open. 2020;3: e2011192. doi: 10.1001/jamanetworkopen.2020.11192 32501489 PMC7275245

[54] Spiezia L, Boscolo A, Poletto F, Cerruti L, Tiberio I, Campello E, et al. COVID-19-Related Severe Hypercoagulability in Patients Admitted to Intensive Care Unit for Acute Respiratory Failure. Thromb Haemost. 2020;120: 998–1000. doi: 10.1055/s-0040-1710018 32316063 PMC7295272

[55] Spiezia L, Campello E, Cola M, Poletto F, Cerruti L, Poretto A, et al. More Severe Hypercoagulable State in Acute COVID-19 Pneumonia as Compared With Other Pneumonia. mcp:iqo. 2020;4: 696–702. doi: 10.1016/j.mayocpiqo.2020.09.002 33024937 PMC7528900

[56] Saseedharan S, Talla VB, Chiluka A. Thromboelastography Profile of Patients with COVID-19 Admitted to Intensive Care Unit: A Single-center Retrospective Study from India. Indian J Crit Care Med. 2020;24: 1218–1222. doi: 10.5005/jp-journals-10071-23675 33446976 PMC7775920

[57] Boscolo A, Spiezia L, Correale C, Sella N, Pesenti E, Beghetto L, et al. Different Hypercoagulable Profiles in Patients with COVID-19 Admitted to the Internal Medicine Ward and the Intensive Care Unit. Thromb Haemost. 2020;120: 1474–1477. doi: 10.1055/s-0040-1714350 32702757

[58] Spiezia L, Boscolo A, Poletto F, Cerruti L, Tiberio I, Campello E, et al. COVID-19-Related Severe Hypercoagulability in Patients Admitted to Intensive Care Unit for Acute Respiratory Failure. Thromb Haemost. 2020;120: 998–1000. doi: 10.1055/s-0040-1710018 32316063 PMC7295272

[59] Wright FL, Vogler TO, Moore EE, Moore HB, Wohlauer MV, Urban S, et al. Fibrinolysis Shutdown Correlation with Thromboembolic Events in Severe COVID-19 Infection. J Am Coll Surg. 2020;231: 193–203.e1. doi: 10.1016/j.jamcollsurg.2020.05.007 32422349 PMC7227511

[60] Görlinger K, Almutawah H, Almutawaa F, Alwabari M, Alsultan Z, Almajed J, et al. The role of rotational thromboelastometry during the COVID-19 pandemic: a narrative review. Korean J Anesthesiol. 2021;74: 91–102. doi: 10.4097/kja.21006 33440114 PMC8024216

[61] Chaudhary R, Kreutz RP, Bliden KP, Tantry US, Gurbel PA. Personalizing Antithrombotic Therapy in COVID-19: Role of Thromboelastography and Thromboelastometry. Thromb Haemost. 2020;120: 1594–1596. doi: 10.1055/s-0040-1714217 32679595

[62] Ibañez C, Perdomo J, Calvo A, Ferrando C, Reverter JC, Tassies D, et al. High D dimers and low global fibrinolysis coexist in COVID19 patients: what is going on in there? J Thromb Thrombolysis. 2021;51: 308–312. doi: 10.1007/s11239-020-02226-0 32671609 PMC7363162

[63] Kruse JM, Magomedov A, Kurreck A, Münch FH, Koerner R, Kamhieh-Milz J, et al. Thromboembolic complications in critically ill COVID-19 patients are associated with impaired fibrinolysis. Critical Care. 2020;24: 676. doi: 10.1186/s13054-020-03401-8 33287877 PMC7719734

[64] Tsantes AE, Frantzeskaki F, Tsantes AG, Rapti E, Rizos M, Kokoris SI, et al. The haemostatic profile in critically ill COVID-19 patients receiving therapeutic anticoagulant therapy: An observational study. Medicine (Baltimore). 2020;99: e23365. doi: 10.1097/MD.0000000000023365 33217881 PMC7676559

[65] Azoulay E, Fartoukh M, Darmon M, Géri G, Voiriot G, Dupont T, et al. Increased mortality in patients with severe SARS-CoV-2 infection admitted within seven days of disease onset. Intensive Care Med. 2020;46: 1714–1722. doi: 10.1007/s00134-020-06202-3 32780165 PMC7417780

[66] Roh DJ, Eiseman K, Kirsch H, Yoh N, Boehme A, Agarwal S, et al. Hypercoagulable viscoelastic blood clot characteristics in critically ill coronavirus disease 2019 patients and associations with thrombotic complications. J Trauma Acute Care Surg. 2021;90: e7–e12. doi: 10.1097/TA.0000000000002963 33009340 PMC8630999

[67] COVID-ICU Group on behalf of the REVA Network and the COVID-ICU Investigators. Clinical characteristics and day-90 outcomes of 4244 critically ill adults with COVID-19: a prospective cohort study. Intensive Care Med. 2021;47: 60–73. doi: 10.1007/s00134-020-06294-x 33211135 PMC7674575

[68] Zhang L, Yan X, Fan Q, Liu H, Liu X, Liu Z, et al. D-dimer levels on admission to predict in-hospital mortality in patients with Covid-19. J Thromb Haemost. 2020;18: 1324–1329. doi: 10.1111/jth.14859 32306492 PMC7264730

[69] Ölander F, Schött U. Monitoring of Low Molecular Weight Heparin Thromboprophylaxis with Alternative Methods to Anti-Factor Xa. Anesthesia and Medical Practice Journal. 2021 [cited 27 Sep 2022]. Available: https://www.gavinpublishers.com/article/view/monitoring-of-low-molecular-weight-heparin-thromboprophylaxis-with-alternative-methods-to-anti-factor-xa.

[70] Schött U, Larsson A, Thomas O, Tynngård N. Monitoring of treatment with low molecular weight heparins using viscoelastic devices. Critical Care. 2014;18: P95. doi: 10.1186/cc13285

[71] Choi JK, Prabhakaran K, Latifi R, Smiley A, Klein J, Lombardo G, et al. Serial rotational thromboelastography (ROTEM) in mechanically ventilated patients with COVID-19 demonstrates hypercoagulopathy despite therapeutic heparinization. Trauma Surg Acute Care Open. 2022;7: e000603. doi: 10.1136/tsaco-2020-000603 35291340 PMC8914399

