# Supplementary material for: Hypercoagulability in critically ill patients with COVID 19, an observational prospective study
Source: PLoS One. 2022 Nov 23;17(11):e0277544. doi: 10.1371/journal.pone.0277544 (PMC9683576; doi:10.1371/journal.pone.0277544)
Supplement: S1 Table — TP: Prothrombin Time; aPTT: Activated Partial Thromboplastin Time; Fg: Fibrinogen; E: EXTEM; I: INTEM; F: FIBTEM; H: HEPTEM; CT: Clotting time; CFT: Clot formation time; A5: Clot amplitude at 5 minutes; MCF: Maximum clot firmness. (DOCX) [file pone.0277544.s001.docx]

Table S 1: Coefficient correlation matrix between indices from ROTEM and others from usual laboratory features

TP: Prothrombin Time; aPTT: Activated Partial Thromboplastin Time; Fg: Fibrinogen; E: EXTEM; I: INTEM; F: FIBTEM; H: HEPTEM; CT : clotting time; CFT : clot formation time; A5 : clot amplitude at 5 minutes; MCF : maximum clot firmness.
