# Supplementary material for: Hypercoagulability in critically ill patients with COVID 19, an observational prospective study
Source: PLoS One. 2022 Nov 23;17(11):e0277544. doi: 10.1371/journal.pone.0277544 (PMC9683576; doi:10.1371/journal.pone.0277544)
Supplement: S2 Table — CRP: C reactive protein; Fg: Fibrinogen; E: EXTEM; CFT: Clot formation time; A5: Clot amplitude at 5 minutes; MCF: Maximum clot firmness; Li60: Lysis index at 60 minutes. (DOCX) [file pone.0277544.s002.docx]

Table S 2: Coefficient correlation matrix between indices of hypercoagulability and inflammatory parameters

|  | CRP | Ferritin | Il1 | Il6 | Il10 | HLA DR | D-Dimers | Fg | E G-score | E CFT | E A5 | E MCF | E Li60 |
| --- | --- | --- | --- | --- | --- | --- | --- | --- | --- | --- | --- | --- | --- |
| CRP | 1 | 0.18 | 0.01 | 0.08 | 0.1 | -0.09 | 0.14 | 0.48 | 0.13 | -0.18 | 0.33 | 0.34 | 0.27 |
| Ferritin | 0.18 | 1 | -0.02 | -0.08 | 0.48 | -0.11 | -0.02 | 0.23 | -0.05 | -0.16 | 0.02 | 0.03 | 0.18 |
| Il1 | 0.01 | -0.02 | 1 | 0.35 | 0.27 | -0.01 | -0.07 | -0.04 | -0.07 | -0.09 | -0.02 | -0.04 | -0.08 |
| Il6 | 0.08 | -0.08 | 0.35 | 1 | 0.19 | -0.07 | -0.03 | 0.03 | -0.04 | -0.04 | -0.08 | -0.06 | 0.12 |
| Il10 | 0.1 | 0.48 | 0.27 | 0.19 | 1 | -0.11 | -0.05 | 0.03 | 0.01 | -0.11 | 0.06 | 0.05 | 0.06 |
| HLA DR | -0.09 | -0.11 | -0.01 | -0.07 | -0.11 | 1 | 0.11 | 0.06 | -0.05 | 0 | -0.04 | -0.08 | -0.21 |
| D-dimers | 0.14 | -0.02 | -0.07 | -0.03 | -0.05 | 0.11 | 1 | -0.14 | -0.03 | 0.23 | -0.12 | -0.04 | 0.23 |
| Fg | 0.48 | 0.23 | -0.04 | 0.03 | 0.03 | 0.06 | -0.14 | 1 | 0.11 | -0.38 | 0.34 | 0.38 | 0.06 |
| E G-score | 0.13 | -0.05 | -0.07 | -0.04 | 0.01 | -0.05 | -0.03 | 0.11 | 1 | -0.28 | 0.35 | 0.36 | 0.14 |
| E CFT | -0.18 | -0.16 | -0.09 | -0.04 | -0.11 | 0 | 0.23 | -0.38 | -0.28 | 1 | -0.78 | -0.71 | -0.15 |
| E A5 | 0.33 | 0.02 | -0.02 | -0.08 | 0.06 | -0.04 | -0.12 | 0.34 | 0.35 | -0.78 | 1 | 0.9 | 0.3 |
| E MCF | 0.34 | 0.03 | -0.04 | -0.06 | 0.05 | -0.08 | -0.04 | 0.38 | 0.36 | -0.71 | 0.9 | 1 | 0.6 |
| E Li60 | 0.27 | 0.18 | -0.08 | 0.12 | 0.06 | -0.21 | 0.23 | 0.06 | 0.14 | -0.15 | 0.3 | 0.6 | 1 |

CRP : C reactive protein; Fg: Fibrinogen; E: EXTEM; CFT: clot formation time; A5 : clot amplitude at 5 minutes; MCF : maximum clot firmness; Li60 : lysis index at 60 minutes.
