# Supplementary material for: Hypercoagulability in critically ill patients with COVID 19, an observational prospective study
Source: PLoS One. 2022 Nov 23;17(11):e0277544. doi: 10.1371/journal.pone.0277544 (PMC9683576; doi:10.1371/journal.pone.0277544)
Supplement: S3 Table — § EXTEM G-score is defined as follows: G-score = (5000xMCF) / (100-MCF). §§ Hypercoagulation due to fibrinogenemia was defined by a higher difference between observed and normal MCF values of FIBTEM than between observed and normal MCF values of EXTEM. §§§ Hypercoagulability were defined by at least one of the following indices: Fibrinogen > 8 g/L, D-Dimers > 3000 μg/dL, EXTEM CFT shorter than normal range, EXTEM A5 higher than normal range, EXTEM MCF higher than normal range, G score ≥11 dyne/cm2, and EXTEM Li 60 > 96.5%. CFT: Clot formation time; A5: Clot amplitude at 5 minutes; MCF: Maximum clot firmness; Li60: Lysis index at 60 minutes. (DOCX) [file pone.0277544.s003.docx]

Table S 3: Comparison between day 1 and day 4 for standard hemostasis and ROTEM

| Variables | Day 1 | Day 4 | Pvalue |
| --- | --- | --- | --- |
| *Number of patients* | N=67 | N=67 |  |
| Standard coagulation tests |  |  |  |
| Platelet (G/L)(missing=1) | 259.6 [190 ; 307] | 307 [249 ; 403] | <0.01 |
| FIBRINOGEN, g/L | 7 [6 ; 8] | 6.6 [5.8 ; 7.6] | 0.01 |
| FIBRINOGEN > 8 g/L | 15 (22.4) | 7 (10.4) | 0.05 |
| D-DIMERS, µg/dL | 1156 [729 ; 2012] | 876 [657 ; 1895] | 0.37 |
| D-DIMERS > 3000 µg/dL | 10 (15) | 11 (16.4) | 0.76 |
| ROTEM variables |  |  |  |
| EXTEM-CFT, sec | 48 [42 ; 56] | 45 [40 ; 51] | 0.04 |
| EXTEM-CFT, sec (<46 (Normal range)) | 31 (46.2) | 39 (58.2) | 0.09 |
| EXTEM-A5, mm | 55 [51 ; 59] | 57 [53 ; 61] | <0.01 |
| EXTEM-A5, mm (> 52 (Normal range)) | 46 (68.6) | 55 (82) | 0.02 |
| EXTEM MCF, mm | 73 [69 ; 75] | 74 [72 ; 77] | <0.01 |
| EXTEM MCF, mm (> 72 (Normal range)) | 41 (61.2) | 53 (79.2) | <0.01 |
| EXTEM G-score § | 13.6 [11.6 ; 15] | 14.2 [12.8 ; 16.8] | <0.01 |
| EXTEM G-score > 11 | 55 (82) | 62 (92.6) | 0.03 |
| EXTEM Li60, % (missing=16) | 97.6 [95.6 ; 99] | 99 [97 ; 100] | <0.01 |
| EXTEM Li60, % (>96.5 (Normal range))(missing=16) | 35 (67.4) | 46 (90.2) | 0.01 |
| Hypercoagulation due to fibrinogenemia§§ | 66 (98.6) | 63 (94) | 0.18 |
| INTEM CT / HEPTEM CT > 1 | 33 (49.2) | 47 (70.2) | <0.01 |
| Presence of at least 1 index in favor of hypercoagulability§§§ | 59 (88) | 66 (98.6) | 0.02 |
| Presence of at least 4 indices in favor of hypercoagulability | 44 (65.6) | 51 (76.2) | 0.04 |

§ EXTEM G-score is defined as follows: G-score = (5000xMCF) / (100-MCF)

§§ Hypercoagulation due to fibrinogenemia was defined by a higher difference between observed and normal MCF values of FIBTEM than between observed and normal MCF values of EXTEM

§§§ Hypercoagulability were defined by at least one of the following indices: fibrinogen > 8 g/L, D-Dimers > 3000 µg/dL, EXTEM CFT shorter than normal range, EXTEM A5 higher than normal range, EXTEM MCF higher than normal range, G score ≥11 dyne/cm2, and EXTEM Li 60 > 96.5%.

CFT : clot formation time; A5 : clot amplitude at 5 minutes; MCF : maximum clot firmness; Li60 : lysis index at 60 minutes
