# Supplementary material for: Hypercoagulability in critically ill patients with COVID 19, an observational prospective study
Source: PLoS One. 2022 Nov 23;17(11):e0277544. doi: 10.1371/journal.pone.0277544 (PMC9683576; doi:10.1371/journal.pone.0277544)
Supplement: S4 Table — TE: Thrombo-embolic event; CFT: Clot formation time; A5: Clot amplitude at 5 minutes; MCF: Maximum clot firmness; Li60: Lysis index at 6 minutes; CRP: C reactive protein. (DOCX) [file pone.0277544.s004.docx]

Table S4: Factors associated with the occurrence of thrombo-embolic events during intensive care stay

|  | Day 1 | |  | Day 4 (65) | | |  | |
| --- | --- | --- | --- | --- | --- | --- | --- | --- |
|  | No TE (N=105) | TE (N=17) | Pval | No TE (N=53) | TE (N=12) | Pval | |  |
| Platelet (G/L)(missing=1) | 247.5 [190.5 ; 311] | 255 [182 ; 304] | 0.99 | 290 [204 ; 405] | 322 [295 ; 396] | 0.16 | |  |
| Platelet > 400 G/L (missing=2) | 11 (10.6) | 1 (5.9) | 0.55 | 26 (26) | 4 (23.5) | 0.83 | |  |
| FIBRINOGEN, g/L | 7 [5.9 ; 7.8] | 7.5 [6.4 ; 7.9] | 0.30 | 6.6 [5.4 ; 7.6] | 6.9 [6.5 ; 7.4] | 0.38 | |  |
| FIBRINOGEN > 8 g/L | 25 (23.6) | 4 (23.5) | 1.00 | 14 (14.9) | 4 (23.5) | 0.37 | |  |
| DDIMER, µg/dL | 1148 [727 ; 1928] | 1349 [642 ; 1841] | 0.98 | 948 [680 ; 1876] | 1504.5 [783 ; 1983.5] | 0.15 | |  |
| DDIMER > 3000 µg/dL | 15 (14.2) | 1 (5.9) | 0.35 | 13 (12.9) | 3 (18.8) | 0.52 | |  |
| EXTEM-CFT, sec | 48 [42 ; 56] | 49 [44 ; 56] | 0.60 | 45 [41 ; 49] | 45.5 [40 ; 54.5] | 0.86 | |  |
| EXTEM-CFT, sec (< Normal range) | 49 (46.2) | 7 (41.2) | 0.70 | 32 (59.3) | 7 (58.3) | 0.95 | |  |
| EXTEM-A5, mm | 55 [50 ; 59] | 53 [51 ; 59] | 0.63 | 57 [53 ; 61] | 57 [53 ; 61] | 0.95 | |  |
| EXTEM-A5, mm (> Normal range) | 73 (68.9) | 10 (58.8) | 0.41 | 44 (81.5) | 10 (83.3) | 0.88 | |  |
| EXTEM MCF, mm | 73 [69 ; 75] | 72 [69 ; 75] | 0.96 | 74 [72 ; 77] | 75.5 [73 ; 78.5] | 0.26 | |  |
| EXTEM MCF, mm (> Normal range) | 62 (58.5) | 10 (58.8) | 0.98 | 42 (77.8) | 10 (83.3) | 0.67 | |  |
| EXTEM G score | 13.5 [11.1 ; 15.8] | 12.9 [11.1 ; 15] | 0.88 | 14.2 [12.9 ; 16.7] | 15.5 [13.5 ; 18.3] | 0.26 | |  |
| EXTEM G score > 11 | 81 (76.4) | 13 (76.5) | 1.00 | 49 (90.7) | 12 (100) | 0.27 | |  |
| EXTEM Li60, % (missing=25) | 98 [95 ; 99] | 97 [94 ; 98] | 0.22 | 99 [97 ; 100] | 100 [98 ; 100] | 0.18 | |  |
| EXTEM Li60, % (> Normal range)(missing=25) | 54 (62.1) | 8 (61.5) | 0.97 | 37 (88.1) | 8 (100) | 0.30 | |  |
| INTEM CT/ HEPTEM CT > 1 | 59 (55.7) | 7 (41.2) | 0.27 | 36 (66.7) | 10 (83.3) | 0.26 | |  |
| At least 1 index in favor of hypercoagulability | 91 (85.8) | 15 (88.2) | 0.79 | 75 (70.8) | 13 (76.5) | 0.63 | |  |
| At least 4 indices in favor of hypercoagulability | 37 (34.9) | 7 (41.2) | 0.62 | 28 (51.9) | 7 (58.3) | 0.68 | |  |
| CRP, (missing = 51) | 94.9 [44.9 ; 174] | 161 [97.6 ; 170] | 0.07 |  |  |  | |  |
| Serum ferritin, mcg/ml (missing = 35) | 874.5 [559 ; 1557] | 952 [491 ; 1257] | 0.71 |  |  |  | |  |
| IL-1Ra, pg/mL (missing = 15) | 0.1 [0 ; 1] | 0 [0 ; 0.8] | 0.62 |  |  |  | |  |
| IL-6, pg/mL (missing = 10) | 28.4 [11.8 ; 55.4] | 61 [34.8 ; 104.2] | 0.01 |  |  |  | |  |
| IL-10, pg/mL (missing = 10) | 3.7 [2 ; 6.2] | 5.6 [3.4 ; 8.5] | 0.09 |  |  |  | |  |
| mHLA DR, pg/mL (missing = 19) | 9758.5 [6608 ; 14007] | 7706.5 [6113 ; 10182] | 0.15 |  |  |  | |  |

TE: thrombo-embolic event; CFT : clot formation time; A5 : clot amplitude at 5 minutes ; MCF : maximum clot firmness ; Li60 : lysis index at 6 minutes; CRP : C reactive protein.
