# Supplementary material for: Hypercoagulability in critically ill patients with COVID 19, an observational prospective study
Source: PLoS One. 2022 Nov 23;17(11):e0277544. doi: 10.1371/journal.pone.0277544 (PMC9683576; doi:10.1371/journal.pone.0277544)
Supplement: S5 Table — AUC: Area under the curve; CFT: Clot formation time; A5: Clot amplitude at 5 minutes; MCF: Maximum clot firmness; Li60: Lysis index at 60 minutes. (DOCX) [file pone.0277544.s005.docx]

Table S 5: Prediction of occurrence of thrombo-embolic events during ICU stay by coagulation indices on days 1 and 4

| Thrombo embolic events | AUC | threshold | specificity | sensitivity | accuracy | tn | tp | fn | fp | npv | ppv | 1-specificity | 1-sensitivity | 1-npv | 1-ppv |
| --- | --- | --- | --- | --- | --- | --- | --- | --- | --- | --- | --- | --- | --- | --- | --- |
| Day 1 |  |  |  |  |  |  |  |  |  |  |  |  |  |  |  |
| Platelet | 0.49 [ 0.28 - 0.69 ] | 254.5 | 0.54 | 0.62 | 0.54 | 62 | 5 | 3 | 53 | 0.95 | 0.09 | 0.46 | 0.38 | 0.05 | 0.91 |
| Fibrinogen | 0.59 [ 0.42 - 0.76 ] | 6.15 | 0.3 | 1 | 0.34 | 34 | 8 | 0 | 81 | 1 | 0.09 | 0.7 | 0 | 0 | 0.91 |
| D-dimers | 0.49 [ 0.25 - 0.72 ] | 1346 | 0.59 | 0.62 | 0.59 | 68 | 5 | 3 | 47 | 0.96 | 0.1 | 0.41 | 0.38 | 0.04 | 0.9 |
| EXTEM CFT | 0.55 [ 0.36 - 0.75 ] | 56.5 | 0.25 | 1 | 0.3 | 29 | 8 | 0 | 86 | 1 | 0.09 | 0.75 | 0 | 0 | 0.91 |
| EXTEM A5 | 0.63 [ 0.43 - 0.83 ] | 58.5 | 0.75 | 0.5 | 0.73 | 86 | 4 | 4 | 29 | 0.96 | 0.12 | 0.25 | 0.5 | 0.04 | 0.88 |
| EXTEM MCF | 0.63 [ 0.44 - 0.83 ] | 74.5 | 0.69 | 0.62 | 0.68 | 79 | 5 | 3 | 36 | 0.96 | 0.12 | 0.31 | 0.38 | 0.04 | 0.88 |
| EXTEM G-score | 0.63 [ 0.44 - 0.83 ] | 14.62 | 0.69 | 0.62 | 0.68 | 79 | 5 | 3 | 36 | 0.96 | 0.12 | 0.31 | 0.38 | 0.04 | 0.88 |
| EXTEM Li60 | 0.6 [ 0.39 - 0.82 ] | 98.5 | 0.29 | 1 | 0.32 | 28 | 4 | 0 | 68 | 1 | 0.06 | 0.71 | 0 | 0 | 0.94 |
| Day 4 |  |  |  |  |  |  |  |  |  |  |  |  |  |  |  |
| Platelet | 0.7 [ 0.58 - 0.83 ] | 294.5 | 0.52 | 1 | 0.55 | 57 | 8 | 0 | 53 | 1 | 0.13 | 0.48 | 0 | 0 | 0.87 |
| Fibrinogen | 0.62 [ 0.41 - 0.82 ] | 6.95 | 0.62 | 0.75 | 0.63 | 64 | 6 | 2 | 39 | 0.97 | 0.13 | 0.38 | 0.25 | 0.03 | 0.87 |
| D-dimers | 0.6 [ 0.39 - 0.81 ] | 1159.5 | 0.57 | 0.62 | 0.57 | 62 | 5 | 3 | 47 | 0.95 | 0.1 | 0.43 | 0.38 | 0.05 | 0.9 |
| EXTEM CFT | 0.47 [ 0.19 - 0.74 ] | 50.5 | 0.78 | 0.43 | 0.74 | 46 | 3 | 4 | 13 | 0.92 | 0.19 | 0.22 | 0.57 | 0.08 | 0.81 |
| EXTEM A5 | 0.6 [ 0.33 - 0.86 ] | 63.5 | 0.88 | 0.43 | 0.83 | 52 | 3 | 4 | 7 | 0.93 | 0.3 | 0.12 | 0.57 | 0.07 | 0.7 |
| EXTEM MCF | 0.71 [ 0.48 - 0.93 ] | 77.5 | 0.8 | 0.57 | 0.77 | 47 | 4 | 3 | 12 | 0.94 | 0.25 | 0.2 | 0.43 | 0.06 | 0.75 |
| EXTEM G-score | 0.71 [ 0.48 - 0.93 ] | 17.23 | 0.8 | 0.57 | 0.77 | 47 | 4 | 3 | 12 | 0.94 | 0.25 | 0.2 | 0.43 | 0.06 | 0.75 |
| EXTEM Li60 | 0.81 [ 0.74 - 0.88 ] | 99.5 | 0.62 | 1 | 0.64 | 29 | 3 | 0 | 18 | 1 | 0.14 | 0.38 | 0 | 0 | 0.86 |

AUC: Area under the curve ; CFT : clot formation time ; A5 : clot amplitude at 5 minutes ; MCF : maximum clot firmness ; Li60 :lysis index at 60 minutes
