# Supplementary material for: Hypercoagulability in critically ill patients with COVID 19, an observational prospective study
Source: PLoS One. 2022 Nov 23;17(11):e0277544. doi: 10.1371/journal.pone.0277544 (PMC9683576; doi:10.1371/journal.pone.0277544)
Supplement: S6 Table — CFT: Clot formation time; A5: Clot amplitude at 5 minutes; MCF: Maximum clot firmness; Li60: Lysis index at 60 minutes. (DOCX) [file pone.0277544.s006.docx]

Table S 6: Factors associated with intubation during ICU stay among patients non intubated on admission.

CFT : clot formation time ; A5 : clot amplitude at 5 minutes ; MCF : maximum clot firmness ; Li60 :lysis index at 60 minutes

|  | Day 1 (N=120) | | | | Day 4 (N=(54) | | | |
| --- | --- | --- | --- | --- | --- | --- | --- | --- |
|  | No IMV | IMV | pvalue | No IMV | | IMV | pvalue |  |
| Number of patients | N=74 | N=29 | . | N=37 | | N=17 |  |  |
| Platelet (G/L)(missing=2) | 270.5 [199 ; 322.5] | 231 [161 ; 304] | 0.14 | 342 [276 ; 433] | | 298 [261 ; 396] | 0.39 |  |
| Platelet > 400 G/L (missing=2) | 10 (13.9) | 2 (6.9) | 0.33 | 24 (35.8) | | 7 (24.1) | 0.26 |  |
| FIBRINOGEN, g/L | 7 [6.2 ; 8] | 7 [6.1 ; 7.8] | 0.64 | 6.7 [5.7 ; 7.5] | | 7 [5.9 ; 8.1] | 0.33 |  |
| FIBRINOGEN > 8 g/L | 19 (25.7) | 7 (24.1) | 0.87 | 5 (7.8) | | 7 (25.9) | 0.02 |  |
| D-DIMERS, µg/dL | 1049 [672 ; 1877] | 955 [682 ; 1494] | 0.74 | 871 [604 ; 1465] | | 1245 [688 ; 2005] | 0.10 |  |
| D-DIMERS > 3000 µg/dL | 11 (14.9) | 2 (6.9) | 0.27 | 7 (10.3) | | 5 (17.2) | 0.34 |  |
| EXTEM-CFT, sec | 47 [40 ; 56] | 52 [45 ; 62] | 0.14 | 45 [41 ; 50] | | 45 [41 ; 51] | 0.75 |  |
| EXTEM-CFT, sec (< Normal range) | 35 (47.3) | 10 (34.5) | 0.24 | 21 (56.8) | | 10 (58.8) | 0.89 |  |
| EXTEM-A5, mm | 54 [51 ; 58] | 54 [48 ; 59] | 0.62 | 58 [53 ; 61] | | 58 [54 ; 62] | 0.75 |  |
| EXTEM-A5, mm (> Normal range) | 49 (66.2) | 17 (58.6) | 0.47 | 31 (83.8) | | 15 (88.2) | 0.67 |  |
| EXTEM MCF, mm | 72.5 [69 ; 75] | 72 [68 ; 76] | 0.97 | 75 [72 ; 77] | | 77 [74 ; 78] | 0.12 |  |
| EXTEM MCF, mm (> Normal range) | 42 (56.8) | 16 (55.2) | 0.88 | 29 (78.4) | | 16 (94.1) | 0.15 |  |
| EXTEM G-score | 13.5 [11.1 ; 15] | 12.9 [10.6 ; 15.8] | 0.85 | 15 [12.9 ; 16.7] | | 16.7 [14.2 ; 17.7] | 0.12 |  |
| EXTEM G-score > 11 | 59 (79.7) | 20 (69) | 0.25 | 36 (97.3) | | 17 (100) | 0.49 |  |
| EXTEM Li60, % (miss=24) | 97 [95 ; 99] | 97 [95 ; 98] | 0.82 | 99 [97 ; 100] | | 100 [98 ; 100] | 0.05 |  |
| EXTEM Li60, % (> Normal range)(missing=24) | 34 (55.7) | 13 (61.9) | 0.62 | 23 (85.2) | | 14 (100) | 0.13 |  |
| INTEM CT / HEPTEM CT > 1 | 32 (43.2) | 17 (58.6) | 0.16 | 22 (59.5) | | 13 (76.5) | 0.22 |  |
| At least 1 index in favor of hypercoagulability | 63 (85.1) | 24 (82.8) | 0.76 | 54 (73) | | 22 (75.9) | 0.76 |  |
| At least 4 indices in favor of hypercoagulability | 25 (33.8) | 10 (34.5) | 0.95 | 19 (51.4) | | 10 (58.8) | 0.61 |  |
| CRP, (missing = 49) | 97.6 [44.3 ; 174] | 158 [83 ; 184] | 0.33 |  | |  |  |  |
| Serum ferritin, mcg/ml (missing = 37) | 1148.5 [699 ; 1944] | 952 [491 ; 1257] | 0.22 |  | |  |  |  |
| IL-1Ra, pg/mL (missing = 14) | 0.1 [0 ; 1] | 0 [0 ; 1.3] | 0.97 |  | |  |  |  |
| IL-6, pg/mL (missing = 9) | 22.9 [9.3 ; 63.8] | 47.7 [27.4 ; 87.6] | 0.02 |  | |  |  |  |
| IL-10, pg/mL (missing = 9) | 3.2 [1.5 ; 5.3] | 5.9 [3.7 ; 10.5] | <.01 |  | |  |  |  |
| mHLA DR, pg/mL (missing = 17) | 10747.5 [7139 ; 14999] | 7740 [6332.7 ; 10258] | 0.02 |  | |  |  |  |
