# Supplementary material for: Hypercoagulability in critically ill patients with COVID 19, an observational prospective study
Source: PLoS One. 2022 Nov 23;17(11):e0277544. doi: 10.1371/journal.pone.0277544 (PMC9683576; doi:10.1371/journal.pone.0277544)
Supplement: S7 Table — AUC: Area under the curve; CFT clot formation time; A5: Clot amplitude at 5 minutes; MCF: Maximum clot formation time; Li60: Lysis index at 60 minutes. (DOCX) [file pone.0277544.s007.docx]

Table S7: Prediction of intubation during ICU stay by coagulation indices on days 1 and 4

| Death and/or IMV | AUC | threshold | specificity | sensitivity | accuracy | tn | tp | fn | fp | npv | ppv | 1-specificity | 1-sensitivity | 1-npv | 1-ppv |
| --- | --- | --- | --- | --- | --- | --- | --- | --- | --- | --- | --- | --- | --- | --- | --- |
| Day 1 |  |  |  |  |  |  |  |  |  |  |  |  |  |  |  |
| Platelet | 0.51 [ 0.39 - 0.63 ] | 575.5 | 0.24 | 0.93 | 0.43 | 17 | 26 | 2 | 54 | 0.89 | 0.32 | 0.76 | 0.07 | 0.11 | 0.68 |
| Fibrinogen | 0.5 [ 0.37 - 0.63 ] | 7.35 | 0.62 | 0.46 | 0.58 | 44 | 13 | 15 | 27 | 0.75 | 0.32 | 0.38 | 0.54 | 0.25 | 0.68 |
| D-dimers | 0.57 [ 0.44 - 0.7 ] | 164.5 | 0.87 | 0.29 | 0.71 | 62 | 8 | 20 | 9 | 0.76 | 0.47 | 0.13 | 0.71 | 0.24 | 0.53 |
| EXTEM CFT | 0.51 [ 0.38 - 0.65 ] | 17.23 | 0.92 | 0.21 | 0.72 | 65 | 6 | 22 | 6 | 0.75 | 0.5 | 0.08 | 0.79 | 0.25 | 0.5 |
| EXTEM A5 | 0.58 [ 0.45 - 0.7 ] | 53.5 | 0.69 | 0.46 | 0.63 | 49 | 13 | 15 | 22 | 0.77 | 0.37 | 0.31 | 0.54 | 0.23 | 0.63 |
| EXTEM MCF | 0.49 [ 0.35 - 0.63 ] | 60.5 | 0.87 | 0.25 | 0.7 | 62 | 7 | 21 | 9 | 0.75 | 0.44 | 0.13 | 0.75 | 0.25 | 0.56 |
| EXTEM G-score | 0.51 [ 0.38 - 0.65 ] | 77.5 | 0.92 | 0.21 | 0.72 | 65 | 6 | 22 | 6 | 0.75 | 0.5 | 0.08 | 0.79 | 0.25 | 0.5 |
| EXTEM Li60 | 0.52 [ 0.38 - 0.66 ] | 96.5 | 0.46 | 0.62 | 0.5 | 27 | 13 | 8 | 32 | 0.77 | 0.29 | 0.54 | 0.38 | 0.23 | 0.71 |
| Day 4 |  |  |  |  |  |  |  |  |  |  |  |  |  |  |  |
| Platelet | 0.63 [ 0.5 - 0.75 ] | 1098.5 | 0.66 | 0.61 | 0.65 | 43 | 17 | 11 | 22 | 0.8 | 0.44 | 0.34 | 0.39 | 0.2 | 0.56 |
| Fibrinogen | 0.58 [ 0.44 - 0.72 ] | 7.85 | 0.9 | 0.31 | 0.72 | 55 | 8 | 18 | 6 | 0.75 | 0.57 | 0.1 | 0.69 | 0.25 | 0.43 |
| D-dimers | 0.55 [ 0.42 - 0.68 ] | 305.5 | 0.63 | 0.57 | 0.61 | 41 | 16 | 12 | 24 | 0.77 | 0.4 | 0.37 | 0.43 | 0.23 | 0.6 |
| EXTEM CFT | 0.65 [ 0.49 - 0.81 ] | 13.19 | 0.32 | 0.94 | 0.51 | 12 | 15 | 1 | 25 | 0.92 | 0.38 | 0.68 | 0.06 | 0.08 | 0.62 |
| EXTEM A5 | 0.48 [ 0.31 - 0.65 ] | 44.5 | 0.59 | 0.5 | 0.57 | 22 | 8 | 8 | 15 | 0.73 | 0.35 | 0.41 | 0.5 | 0.27 | 0.65 |
| EXTEM MCF | 0.54 [ 0.37 - 0.71 ] | 63.5 | 0.89 | 0.25 | 0.7 | 33 | 4 | 12 | 4 | 0.73 | 0.5 | 0.11 | 0.75 | 0.27 | 0.5 |
| EXTEM G-score | 0.65 [ 0.49 - 0.81 ] | 72.5 | 0.32 | 0.94 | 0.51 | 12 | 15 | 1 | 25 | 0.92 | 0.38 | 0.68 | 0.06 | 0.08 | 0.62 |
| EXTEM Li60 | 0.7 [ 0.54 - 0.85 ] | 99.5 | 0.67 | 0.69 | 0.68 | 18 | 9 | 4 | 9 | 0.82 | 0.5 | 0.33 | 0.31 | 0.18 | 0.5 |

AUC : area under the curve ; CFT clot formation time ; A5 : clot amplitude at 5 minutes ; MCF : maximum clot formation time ; Li60 :lysis index at 60 minutes.
