# Supplementary material for: Hypercoagulability in critically ill patients with COVID 19, an observational prospective study
Source: PLoS One. 2022 Nov 23;17(11):e0277544. doi: 10.1371/journal.pone.0277544 (PMC9683576; doi:10.1371/journal.pone.0277544)
Supplement: S8 Table — CFT: Clot formation time; A5: Clot amplitude at 5 minutes; MCF: Maximum clot firmness; Li60: Lysis index at 60 minutes. (DOCX) [file pone.0277544.s008.docx]

Table S 8: Comparison of patients who experienced hospital death and those who did not

|  | Day 1 (N=133) | | | | Day 4 (N=67) | | | |
| --- | --- | --- | --- | --- | --- | --- | --- | --- |
|  | No Death | Death | pvalue | No Death | | Death | pvalue |  |
|  | N=87 | N=46 |  | N=42 | | N=25 |  |  |
| Platelet (G/L)(missing=2) | 264 [200 ; 315] | 229 [161 ; 300] | 0.13 | 317.5 [237.5 ; 419.5] | | 289 [230 ; 378] | 0.15 |  |
| Platelet > 400 G/L (missing=2) | 10 (11.8) | 4 (8.7) | 0.59 | 25 (31.3) | | 8 (17.4) | 0.09 |  |
| FIBRINOGEN, g/L | 7 [6 ; 7.9] | 7 [5.9 ; 7.8] | 0.63 | 6.6 [5.6 ; 7.5] | | 6.6 [5.6 ; 8.1] | 0.51 |  |
| FIBRINOGEN > 8 g/L | 21 (24.1) | 11 (23.9) | 0.98 | 7 (9.2) | | 11 (25.6) | 0.02 |  |
| D-DIMERS, µg/dL | 1175 [672 ; 1928] | 1209 [813 ; 2407] | 0.35 | 922.5 [643 ; 1676] | | 1694 [751 ; 2961] | 0.02 |  |
| D-DIMERS > 3000 µg/dL | 14 (16.1) | 6 (13) | 0.64 | 8 (10) | | 11 (23.9) | 0.04 |  |
| EXTEM-CFT, sec | 47 [41 ; 56] | 49.5 [42 ; 62] | 0.22 | 45 [41 ; 49] | | 46 [40 ; 55] | 0.44 |  |
| EXTEM-CFT, sec (< Normal range) | 43 (49.4) | 18 (39.1) | 0.26 | 25 (59.5) | | 14 (56) | 0.78 |  |
| EXTEM-A5, mm | 55 [51 ; 58] | 55.5 [48 ; 60] | 0.92 | 57 [53 ; 61] | | 57 [53 ; 62] | 1.00 |  |
| EXTEM-A5, mm (> Normal range) | 59 (67.8) | 30 (65.2) | 0.76 | 35 (83.3) | | 20 (80) | 0.73 |  |
| EXTEM MCF, mm | 73 [69 ; 75] | 73 [68 ; 76] | 0.51 | 74 [72 ; 77] | | 74 [73 ; 78] | 0.26 |  |
| EXTEM MCF, mm (> Normal range) | 50 (57.5) | 28 (60.9) | 0.71 | 32 (76.2) | | 21 (84) | 0.45 |  |
| EXTEM G-score | 13.5 [11.1 ; 15] | 13.5 [10.6 ; 15.8] | 0.62 | 14.2 [12.9 ; 16.7] | | 14.2 [13.5 ; 17.7] | 0.26 |  |
| EXTEM G-score > 11 | 68 (78.2) | 34 (73.9) | 0.58 | 40 (95.2) | | 22 (88) | 0.28 |  |
| EXTEM Li60, % (miss=25) | 97 [95 ; 98] | 98 [96 ; 99] | 0.05 | 98 [97 ; 100] | | 100 [98 ; 100] | 0.03 |  |
| EXTEM Li60, % (> Normal range)(missing=25) | 44 (60.3) | 24 (68.6) | 0.40 | 24 (82.8) | | 22 (100) | 0.04 |  |
| INTEM CT / HEPTEM CT > 1 | 40 (46) | 29 (63) | 0.06 | 27 (64.3) | | 20 (80) | 0.17 |  |
| At least 1 index in favor of hypercoagulability | 76 (87.4) | 39 (84.8) | 0.68 | 61 (70.1) | | 33 (71.7) | 0.84 |  |
| At least 4 indices in favor of hypercoagulability | 32 (36.8) | 17 (37) | 0.98 | 20 (47.6) | | 15 (60) | 0.33 |  |
| CRP, (missing = 57) | 102 [45.5 ; 175] | 101 [66 ; 179.5] | 0.63 |  | |  |  |  |
| Serum ferritin, mcg/ml (missing = 37) | 936 [614 ; 1944] | 952 [512 ; 1322] | 0.78 |  | |  |  |  |
| IL-1Ra, pg/mL (missing = 16) | 0.2 [0 ; 1] | 0.1 [0 ; 1.2] | 0.91 |  | |  |  |  |
| IL-6, pg/mL (missing = 11) | 25.6 [10.1 ; 68] | 44.9 [27.2 ; 71.7] | 0.06 |  | |  |  |  |
| IL-10, pg/mL (missing = 11) | 3.2 [1.5 ; 5.2] | 5.8 [3.6 ; 10.5] | <0.01 |  | |  |  |  |
| mHLA DR, pg/mL (missing = 21) | 10472 [7003 ; 15644] | 7728 [6332.7 ; 11403] | 0.02 |  | |  |  |  |

CFT : clot formation time ; A5 : clot amplitude at 5 minutes ; MCF : maximum clot firmness ; Li60 :lysis index at 60 minutes
