# Supplementary material for: Hypercoagulability in critically ill patients with COVID 19, an observational prospective study
Source: PLoS One. 2022 Nov 23;17(11):e0277544. doi: 10.1371/journal.pone.0277544 (PMC9683576; doi:10.1371/journal.pone.0277544)
Supplement: S9 Table — AUC: Area under the curve; CFT clot formation time; A5: Clot amplitude at 5 minutes; MCF: Maximum clot formation time; Li60: Lysis index at 60 minutes. (DOCX) [file pone.0277544.s009.docx]

Table S7: Prediction of occurrence of death by coagulation indices on days 1 and 4

| Death and/or IMV | AUC | threshold | specificity | sensitivity | accuracy | tn | tp | fn | fp | npv | ppv | 1-specificity | 1-sensitivity | 1-npv | 1-ppv |
| --- | --- | --- | --- | --- | --- | --- | --- | --- | --- | --- | --- | --- | --- | --- | --- |
| Day 1 |  |  |  |  |  |  |  |  |  |  |  |  |  |  |  |
| Platelet | 0.58 [ 0.47 - 0.69 ] | 196.5 | 0.78 | 0.39 | 0.65 | 68 | 18 | 28 | 19 | 0.71 | 0.49 | 0.22 | 0.61 | 0.29 | 0.51 |
| Fibrinogen | 0.47 [ 0.37 - 0.58 ] | 7.25 | 0.59 | 0.46 | 0.54 | 51 | 21 | 25 | 36 | 0.67 | 0.37 | 0.41 | 0.54 | 0.33 | 0.63 |
| D-dimers | 0.55 [ 0.45 - 0.65 ] | 678 | 0.26 | 0.89 | 0.48 | 23 | 41 | 5 | 64 | 0.82 | 0.39 | 0.74 | 0.11 | 0.18 | 0.61 |
| EXTEM CFT | 0.57 [ 0.46 - 0.67 ] | 53.5 | 0.71 | 0.43 | 0.62 | 62 | 20 | 26 | 25 | 0.7 | 0.44 | 0.29 | 0.57 | 0.3 | 0.56 |
| EXTEM A5 | 0.51 [ 0.39 - 0.62 ] | 58.5 | 0.76 | 0.33 | 0.61 | 66 | 15 | 31 | 21 | 0.68 | 0.42 | 0.24 | 0.67 | 0.32 | 0.58 |
| EXTEM MCF | 0.54 [ 0.43 - 0.64 ] | 75.5 | 0.8 | 0.33 | 0.64 | 70 | 15 | 31 | 17 | 0.69 | 0.47 | 0.2 | 0.67 | 0.31 | 0.53 |
| EXTEM G-score | 0.54 [ 0.43 - 0.64 ] | 15.42 | 0.8 | 0.33 | 0.64 | 70 | 15 | 31 | 17 | 0.69 | 0.47 | 0.2 | 0.67 | 0.31 | 0.53 |
| EXTEM Li60 | 0.62 [ 0.5 - 0.73 ] | 98.5 | 0.77 | 0.43 | 0.66 | 56 | 15 | 20 | 17 | 0.74 | 0.47 | 0.23 | 0.57 | 0.26 | 0.53 |
| Day 4 |  |  |  |  |  |  |  |  |  |  |  |  |  |  |  |
| Platelet | 0.58 [ 0.47 - 0.68 ] | 305 | 0.58 | 0.63 | 0.6 | 47 | 29 | 17 | 34 | 0.73 | 0.46 | 0.42 | 0.37 | 0.27 | 0.54 |
| Fibrinogen | 0.54 [ 0.42 - 0.65 ] | 7.85 | 0.89 | 0.28 | 0.67 | 68 | 12 | 31 | 8 | 0.69 | 0.6 | 0.11 | 0.72 | 0.31 | 0.4 |
| D-dimers | 0.63 [ 0.52 - 0.73 ] | 1647 | 0.75 | 0.52 | 0.67 | 60 | 24 | 22 | 20 | 0.73 | 0.55 | 0.25 | 0.48 | 0.27 | 0.45 |
| EXTEM CFT | 0.56 [ 0.41 - 0.71 ] | 50.5 | 0.81 | 0.36 | 0.64 | 34 | 9 | 16 | 8 | 0.68 | 0.53 | 0.19 | 0.64 | 0.32 | 0.47 |
| EXTEM A5 | 0.5 [ 0.35 - 0.65 ] | 63.5 | 0.9 | 0.24 | 0.66 | 38 | 6 | 19 | 4 | 0.67 | 0.6 | 0.1 | 0.76 | 0.33 | 0.4 |
| EXTEM MCF | 0.58 [ 0.43 - 0.73 ] | 77.5 | 0.83 | 0.36 | 0.66 | 35 | 9 | 16 | 7 | 0.69 | 0.56 | 0.17 | 0.64 | 0.31 | 0.44 |
| EXTEM G-score | 0.58 [ 0.43 - 0.73 ] | 17.23 | 0.83 | 0.36 | 0.66 | 35 | 9 | 16 | 7 | 0.69 | 0.56 | 0.17 | 0.64 | 0.31 | 0.44 |
| EXTEM Li60 | 0.68 [ 0.54 - 0.82 ] | 97.5 | 0.38 | 0.91 | 0.61 | 11 | 20 | 2 | 18 | 0.85 | 0.53 | 0.62 | 0.09 | 0.15 | 0.47 |

AUC : area under the curve ; CFT clot formation time ; A5 : clot amplitude at 5 minutes ; MCF : maximum clot formation time ; Li60 :lysis index at 60 minutes.
