# Supplementary material for: Hypercoagulability in critically ill patients with COVID 19, an observational prospective study
Source: PLoS One. 2022 Nov 23;17(11):e0277544. doi: 10.1371/journal.pone.0277544 (PMC9683576; doi:10.1371/journal.pone.0277544)
Supplement: S10 Table — CFT: Clot formation time; A5: Clot amplitude at 5 minutes; MCF: Maximum clot firmness; Li60: Lysis index at 60 minutes. (DOCX) [file pone.0277544.s010.docx]

Table S 10: Comparison of patients who experienced hospital death and/or invasive mechanical ventilation and those who did not

|  | Day 1 (N=133) | | | | Day 4 (N=67) | | | |
| --- | --- | --- | --- | --- | --- | --- | --- | --- |
|  | No IMV/No Death | Death and/or IMV | pvalue | No IVM/No Death | | Death and/or IMV | pvalue |  |
| Number of patients | N=74 | N=59 |  | N=37 | | N=30 |  |  |
| Platelet (G/L)(missing=2) | 270.5 [199 ; 322.5] | 229 [179 ; 304] | 0.15 | 342 [276 ; 433] | | 296 [240 ; 393] | 0.15 |  |
| Platelet > 400 G/L (missing=2) | 10 (13.9) | 4 (9.5) | 0.49 | 24 (35.8) | | 9 (21.4) | 0.11 |  |
| FIBRINOGEN, g/L | 7 [6.2 ; 8] | 7.1 [5.9 ; 7.8] | 0.55 | 6.7 [5.7 ; 7.5] | | 6.6 [5.4 ; 8] | 0.99 |  |
| FIBRINOGEN > 8 g/L | 19 (25.7) | 10 (23.8) | 0.82 | 5 (7.8) | | 10 (25) | 0.02 |  |
| D-DIMERS, µg/dL | 1049 [672 ; 1877] | 1146.5 [711 ; 2012] | 0.67 | 871 [604 ; 1465] | | 1260 [688 ; 2593] | 0.04 |  |
| D-DIMERS > 3000 µg/dL | 11 (14.9) | 4 (9.5) | 0.41 | 7 (10.3) | | 8 (19) | 0.19 |  |
| EXTEM-CFT, sec | 47 [40 ; 56] | 49.5 [43 ; 62] | 0.22 | 45 [41 ; 50] | | 45.5 [40 ; 55] | 0.52 |  |
| EXTEM-CFT, sec (< Normal range) | 35 (47.3) | 16 (38.1) | 0.34 | 21 (56.8) | | 12 (54.5) | 0.87 |  |
| EXTEM-A5, mm | 54 [51 ; 58] | 55.5 [49 ; 60] | 0.87 | 58 [53 ; 61] | | 57 [53 ; 62] | 0.85 |  |
| EXTEM-A5, mm (> Normal range) | 49 (66.2) | 28 (66.7) | 0.96 | 31 (83.8) | | 18 (81.8) | 0.85 |  |
| EXTEM MCF, mm | 72.5 [69 ; 75] | 73 [68 ; 76] | 0.56 | 75 [72 ; 77] | | 74.5 [73 ; 78] | 0.43 |  |
| EXTEM MCF, mm (> Normal range) | 42 (56.8) | 26 (61.9) | 0.59 | 29 (78.4) | | 19 (86.4) | 0.45 |  |
| EXTEM G-score | 13.5 [11.1 ; 15] | 13.5 [10.6 ; 15.8] | 0.70 | 15 [12.9 ; 16.7] | | 14.6 [13.5 ; 17.7] | 0.43 |  |
| EXTEM G-score > 11 | 59 (79.7) | 31 (73.8) | 0.46 | 36 (97.3) | | 20 (90.9) | 0.28 |  |
| EXTEM Li60, % (miss=24) | 97 [95 ; 99] | 98 [96 ; 99] | 0.26 | 99 [97 ; 100] | | 100 [98 ; 100] | 0.03 |  |
| EXTEM Li60, % (> Normal range)(missing=24) | 34 (55.7) | 21 (67.7) | 0.27 | 23 (85.2) | | 19 (100) | 0.08 |  |
| INTEM CT / HEPTEM CT > 1 | 32 (43.2) | 27 (64.3) | 0.03 | 22 (59.5) | | 17 (77.3) | 0.16 |  |
| At least 1 index in favor of hypercoagulability | 63 (85.1) | 36 (85.7) | 0.93 | 54 (73) | | 29 (69) | 0.65 |  |
| At least 4 indices in favor of hypercoagulability | 25 (33.8) | 16 (38.1) | 0.64 | 19 (51.4) | | 13 (59.1) | 0.56 |  |
| CRP, (missing = 49) | 97.6 [44.3 ; 174] | 114 [67.5 ; 179.5] | 0.44 |  | |  |  |  |
| Serum ferritin, mcg/ml (missing = 37) | 1148.5 [699 ; 1944] | 857.5 [489 ; 1322] | 0.21 |  | |  |  |  |
| IL-1Ra, pg/mL (missing = 14) | 0.1 [0 ; 1] | 0 [0 ; 1.2] | 0.84 |  | |  |  |  |
| IL-6, pg/mL (missing = 9) | 22.9 [9.3 ; 63.8] | 44.5 [19.6 ; 71.7] | 0.09 |  | |  |  |  |
| IL-10, pg/mL (missing = 9) | 3.2 [1.5 ; 5.3] | 5.5 [3.6 ; 10.5] | <0.01 |  | |  |  |  |
| mHLA DR, pg/mL (missing = 17) | 10747.5 [7139 ; 14999] | 7685 [6332.7 ; 10182] | <0.01 |  | |  |  |  |

CFT : clot formation time ; A5 : clot amplitude at 5 minutes ; MCF : maximum clot firmness ; Li60 :lysis index at 60 minutes
