# Supplementary material for: Hypercoagulability in critically ill patients with COVID 19, an observational prospective study
Source: PLoS One. 2022 Nov 23;17(11):e0277544. doi: 10.1371/journal.pone.0277544 (PMC9683576; doi:10.1371/journal.pone.0277544)
Supplement: S11 Table — AUC: Area under the curve; CFT clot formation time; A5: Clot amplitude at 5 minutes; MCF: Maximum clot formation time; Li60: Lysis index at 60 minutes. (DOCX) [file pone.0277544.s011.docx]

Table S11: Prediction of occurrence of death and/or intubation by coagulation indices on days 1 and 4

| Death and/or IMV | AUC | threshold | specificity | sensitivity | accuracy | tn | tp | fn | fp | npv | ppv | 1-specificity | 1-sensitivity | 1-npv | 1-ppv |
| --- | --- | --- | --- | --- | --- | --- | --- | --- | --- | --- | --- | --- | --- | --- | --- |
| Day 1 |  |  |  |  |  |  |  |  |  |  |  |  |  |  |  |
| Platelet | 0.56 [ 0.46 - 0.67 ] | 305.5 | 0.32 | 0.81 | 0.54 | 21 | 46 | 11 | 45 | 0.66 | 0.51 | 0.68 | 0.19 | 0.34 | 0.49 |
| Fibrinogen | 0.45 [ 0.35 - 0.56 ] | 7.05 | 0.55 | 0.49 | 0.52 | 36 | 28 | 29 | 30 | 0.55 | 0.48 | 0.45 | 0.51 | 0.45 | 0.52 |
| D-dimers | 0.61 [ 0.51 - 0.71 ] | 1195.5 | 0.62 | 0.56 | 0.59 | 41 | 32 | 25 | 25 | 0.62 | 0.56 | 0.38 | 0.44 | 0.38 | 0.44 |
| EXTEM CFT | 0.58 [ 0.48 - 0.68 ] | 48.5 | 0.61 | 0.53 | 0.57 | 40 | 30 | 27 | 26 | 0.6 | 0.54 | 0.39 | 0.47 | 0.4 | 0.46 |
| EXTEM A5 | 0.51 [ 0.4 - 0.62 ] | 60.5 | 0.88 | 0.26 | 0.59 | 58 | 15 | 42 | 8 | 0.58 | 0.65 | 0.12 | 0.74 | 0.42 | 0.35 |
| EXTEM MCF | 0.54 [ 0.43 - 0.65 ] | 75.5 | 0.83 | 0.33 | 0.6 | 55 | 19 | 38 | 11 | 0.59 | 0.63 | 0.17 | 0.67 | 0.41 | 0.37 |
| EXTEM G-score | 0.54 [ 0.43 - 0.65 ] | 15.42 | 0.83 | 0.33 | 0.6 | 55 | 19 | 38 | 11 | 0.59 | 0.63 | 0.17 | 0.67 | 0.41 | 0.37 |
| EXTEM Li60 | 0.6 [ 0.49 - 0.71 ] | 95.5 | 0.36 | 0.82 | 0.57 | 20 | 37 | 8 | 35 | 0.71 | 0.51 | 0.64 | 0.18 | 0.29 | 0.49 |
| Day 4 |  |  |  |  |  |  |  |  |  |  |  |  |  |  |  |
| Platelet | 0.62 [ 0.52 - 0.72 ] | 305.5 | 0.61 | 0.67 | 0.64 | 37 | 38 | 19 | 24 | 0.66 | 0.61 | 0.39 | 0.33 | 0.34 | 0.39 |
| Fibrinogen | 0.49 [ 0.38 - 0.6 ] | 4.85 | 0.95 | 0.21 | 0.59 | 55 | 11 | 42 | 3 | 0.57 | 0.79 | 0.05 | 0.79 | 0.43 | 0.21 |
| D-dimers | 0.68 [ 0.59 - 0.78 ] | 1612.5 | 0.84 | 0.52 | 0.68 | 51 | 29 | 27 | 10 | 0.65 | 0.74 | 0.16 | 0.48 | 0.35 | 0.26 |
| EXTEM CFT | 0.54 [ 0.4 - 0.69 ] | 37.5 | 0.22 | 0.93 | 0.55 | 8 | 28 | 2 | 28 | 0.8 | 0.5 | 0.78 | 0.07 | 0.2 | 0.5 |
| EXTEM A5 | 0.56 [ 0.42 - 0.71 ] | 58.5 | 0.5 | 0.73 | 0.61 | 18 | 22 | 8 | 18 | 0.69 | 0.55 | 0.5 | 0.27 | 0.31 | 0.45 |
| EXTEM MCF | 0.5 [ 0.36 - 0.65 ] | 74.5 | 0.53 | 0.6 | 0.56 | 19 | 18 | 12 | 17 | 0.61 | 0.51 | 0.47 | 0.4 | 0.39 | 0.49 |
| EXTEM G-score | 0.5 [ 0.36 - 0.65 ] | 14.62 | 0.53 | 0.6 | 0.56 | 19 | 18 | 12 | 17 | 0.61 | 0.51 | 0.47 | 0.4 | 0.39 | 0.49 |
| EXTEM Li60 | 0.64 [ 0.49 - 0.79 ] | 97.5 | 0.38 | 0.88 | 0.62 | 10 | 21 | 3 | 16 | 0.77 | 0.57 | 0.62 | 0.12 | 0.23 | 0.43 |

AUC : area under the curve ; CFT clot formation time ; A5 : clot amplitude at 5 minutes ; MCF : maximum clot formation time ; Li60 :lysis index at 60 minutes.
