# Supplementary material for: Hypercoagulability in critically ill patients with COVID 19, an observational prospective study
Source: PLoS One. 2022 Nov 23;17(11):e0277544. doi: 10.1371/journal.pone.0277544 (PMC9683576; doi:10.1371/journal.pone.0277544)
Supplement: S12 Table — HDPA: High dose preventive anticoagulation; CDA: Curative dose anticoagulation. (DOCX) [file pone.0277544.s012.docx]

Table S12: Anticoagulation and prolonged CT on day 4

| At day 4 | INTEM/HEPTEM CT < 1 | INTEM/HEPTEM CT > 1 | Pval |
| --- | --- | --- | --- |
| No anticoagulation | 2 (11.1) | 2 (4.5) | 0.02 |
| LMWH Preventive | 0 (0) | 1 (2.3) | . |
| LMWH HDPA | 13 (72.2) | 15 (34.1) | . |
| LMWH CDA | 2 (11.1) | 13 (29.5) | . |
| UFH HDPA | 0 (0) | 3 (6.8) | . |
| UFH CDA | 0 (0) | 10 (22.7) | . |
| Direct Oral Anticoagulants | 1 (5.6) | 0 (0) | . |
| Anti-vitamin K | 0 (0) | 0 (0) | . |

HDPA: high dose preventive anticoagulation; CDA: curative dose anticoagulation
