# Supplementary material for: Hypercoagulability in critically ill patients with COVID 19, an observational prospective study
Source: PLoS One. 2022 Nov 23;17(11):e0277544. doi: 10.1371/journal.pone.0277544 (PMC9683576; doi:10.1371/journal.pone.0277544)
Supplement: S1 File — (DOCX) [file pone.0277544.s013.docx]

**SUPPLEMENTAL FILES**

**Definitions**

**Rotational thrombo-elastometry (ROTEM)**

Rotational thrombo-elastometry (ROTEM) provide comprehensive, dynamic and timely assessment of clot initiation, propagation, clot quality and stability in whole blood. A tube of blood is rotated around a central probe that detects various clotting parameters, including clotting time, speed of clot formation, clot strength and characteristics of fibrinolysis. Clot formation is activated by addition of different drivers to analyze different aspects of coagulation (extrinsically activated assays using tissue factor (EXTEM), intrinsically activated assays using phospholipid and ellagic acid (INTEM), fibrin-based extrinsically activated assays using tissue factor and platelet inhibitor cytochalasin D (FIBTEM), intrinsically activated assays with the addition of heparinase for heparin impact on coagulation (HEPTEM). EXTEM and INTEM provide information about the extrinsic and intrinsic coagulation pathways, respectively. FIBTEM blocks the platelet contribution to clot formation, leaving only the impact of fibrin formation and polymerization. HEPTEM presents coagulation disturbances where the effects of heparin are excluded.

Coagulation Time (CT) is the time (in seconds) from test start until an amplitude of 2 mm is reached, giving information about coagulation activation/initiation. Clot Formation Time (CFT) is the time (in seconds) between 2 mm amplitude and 20 mm amplitude, giving information about clot propagation. Maximum Clot Firmness (MCF) is the maximum amplitude (in millimeters) reached during the

test, giving information about clot stability. A5 represent clot firmness (in millimeters) after 5 min. LI-60 is the reduction in MCF 60 min after CT (in percent).

**Anticoagulation**

***Standard prophylactic anticoagulation*** was defined by the administration of enoxaparin 4000 Ui/ 24h.

***High dose prophylactic anticoagulation*** was defined by higher doses than the standard prophylactic regimen: enoxaparin 6000 Ui/ 24 h if body mass index (BMI) was below 30 kg/m², enoxaparin 4000 UI/12 h if BMI was between 30 and 35 kg/m², and enoxaparin 6000 Ui/12 h if BMI was above 35 kg/m². Anti-Xa monitoring was performed if clearance was below 20 ml/min and if BMI was above 40 kg/m², in order to avoid overdose.

***Curative dose anticoagulation*** (CDA) was defined by administration of enoxaparin 100 IU/kg/12h or unfractionated heparin 500 IU/kg/24h.

**Bleeding**

***Major bleeding*** was defined as acute bleeding with one or more of the following: fatal bleeding, symptomatic or clinically manifest bleeding in a critical area or organ (intracranial, intraspinal, intraocular, retroperitoneal, intraarticular, pericardial, or intramuscular with compartment syndrome), bleeding causing a fall in haemoglobin of ≥ 2g/dl, or leading to the transfusion of two or more whole blood or red cells, according to ISTH guidelines (Schulman S, J Thromb Haemost. 2005;3:692-694).
