# Supplementary material for: Hypercoagulability in critically ill patients with COVID 19, an observational prospective study
Source: PLoS One. 2022 Nov 23;17(11):e0277544. doi: 10.1371/journal.pone.0277544 (PMC9683576; doi:10.1371/journal.pone.0277544)
Supplement: Attachment — Submitted filename: ELASTO COVID Response to reviewers.docx [file pone.0277544.s015.docx]

**Response to reviewers:**

ACADEMIC EDITOR:

Dear authors,
the paper looks interesting and may add a further step in understanding the negative impact of the hypercoagulable state found in critically ill patients with COVID-19. I think it is a good paper, well written, but in need of minor revisions as requested by the Reviewers, especially the review of the outcome as per Reviewer #1.

**As requested, we analyzed separately the alteration of coagulation profile according to (1) thromboembolic events, (2) need for intubation, (3) ICU death, and (4) the composite outcome of intubation and/or death.**

Since in several patients withholding of life support resulted in ICU death without intubation, in the analysis of the relationship between coagulation parameters and the need for intubation, the patients who died and were not intubated were excluded.

This is indicated at the end of the introduction section:

“ROTEM tests for predicting TE events, and the unfavorable outcomes including need for intubation, ICU death, and the composite end point of need for intubation and/or ICU mortality”

**In the Statistical analysis**

“Factors associated with (1) the occurrence of TE events among patients free of TE events on admission, (2) intubation (excluding patients who died without intubation because of withholding of life support), (3) ICU death, and (4) the composite outcome including ICU death and/or intubation were investigated using Student or Wilcoxon tests, and the Chi2 or Fisher exact tests for continuous and categorical data, respectively, depending on the distribution of the data.

The ability of the coagulation indices to predict (1) TE events, (2) intubation, (3) ICU death, and (4) ICU death and/or intubation was assessed using the ROC curve.”

**In the results section**

“Factors associated with intubation

The factors associated on day 1 with an increased risk of intubation were…

All the indices measured on days 1 or 4 had a very low ability (all AUC ROC curves < 0.7) to predict the need for intubation (Fig 4, S7 Table).

Factors associated with ICU death

The factors associated on day 1 with an increased risk of ICU death was EXTEM Li60 (p=0.05), and on day 4 higher were serum values of fibrinogen (p=0.02), D-dimers (p = 0.04), and EXTEM Li 60 (p = 0.03). On day 1, higher serum IL-6 and Il-10 values and lower mHLA-DR values were associated with ICU death (S8 Table). All the indices measured on day 1 or 4 had a very low ability (all AUC ROC curves < 0.7) to predict the occurrence of ICU death (Fig 5, S9 Table).

As far as the thromboelastogram is concerned, in addition to a small and brief explanation (no more than 1-2 sentences), it would be useful to include in the main paper a meaningful picture of the ROTEM, showing at a glance the picture that is most frequently seen in these patients.

Thank you for this remark.

First, we added this sentence in the introduction

“Rotational thromboelastometry (ROTEM) is a relocated laboratory assay providing a comprehensive analysis of dynamic and timely changes in the viscoelastic properties of clot formation, including clot initiation, propagation, strengthening, and dissolution. ROTEM tests assess both coagulation and fibrinolysis, are performed on whole blood and therefore take into account the contribution of blood cells. In ROTEM tests, clot formation can be triggered in various ways by the addition of different activators, defining different assays: EXTEM to assess the extrinsic pathway, INTEM the intrinsic pathway, FIBTEM the effect of fibrinogen on coagulation, and HEPTEM that of heparin on coagulation. In ROTEM assays, a probe is suspended within a sample of whole citrated blood, and changes in blood viscosity, which develop while the sample is rotated, are graphically transmitted. (S File). (S File).”

As requested, a meaningful picture of the ROTEM, showing at a glance the picture that is frequently observed in these patients has been included in the paper (Figure 2)

In the results section:

Figure 2 shows the results of ROTEM tests performed on admission in a patient with the graphical shape frequently observed showing both hypercoagulability and hypofibrinolysis.

**Fig 2: Results of ROTEM tests with the graphical shape performed on admission showing both hypercoagulability and hypofibrinolysis.**

CT: clotting time; CFT: clot formation time; A5 – A10 – A20: clot amplitude 5 – 10 – 20 minutes after CT; MCF: maximum clot firmness

In this patient, hypercoagulability was characterized by an EXTEM CFT result below normal range values (< 46s), and EXTEM A5 and EXTEM MCF results above normal range values (> 52 mm and > 72 mm, respectively); hypofibrinolysis was characterized by EXTEM Li 60 > 96.5%.

==============================

Journal requirements:

When submitting your revision, we need you to address these additional requirements.

1. Please ensure that your manuscript meets PLOS ONE's style requirements, including those for file naming. The PLOS ONE style templates can be found at 
https://journals.plos.org/plosone/s/file?id=wjVg/PLOSOne_formatting_sample_main_body.pdf and 
https://journals.plos.org/plosone/s/file?id=ba62/PLOSOne_formatting_sample_title_authors_affiliations.pdf

Ok done

We note that you have provided funding information that is not currently declared in your Funding Statement. However, funding information should not appear in the Acknowledgments section or other areas of your manuscript. We will only publish funding information present in the Funding Statement section of the online submission form. 
Please remove any funding-related text from the manuscript and let us know how you would like to update your Funding Statement. Currently, your Funding Statement reads as follows:

"This study was supported by a grant from Michelin Corporate Foundation.
The funders had no role in study design, data collection and analysis, decision to publish, or preparation of the manuscript."

Ok, thank you for this suggestion. We leave it to you to place the above sentence appropriately in the manuscript.

Please include your amended statements within your cover letter; we will change the online submission form on your behalf.

🡪 We have left our sentence in the Funding paragraph of the manuscript and added **this sentence** in the cover letter.

🡪 We have removed from the Acknowledgements section the sentence about the Michelin Corporation

3. We note that you have indicated that data from this study are available upon request. PLOS only allows data to be available upon request if there are legal or ethical restrictions on sharing data publicly. For more information on unacceptable data access restrictions, please see http://journals.plos.org/plosone/s/data-availability#loc-unacceptable-data-access-restrictions.

OK, all the data are now available publicly.

Reviewers' comments:

Reviewer's Responses to Questions

**Comments to the Author**

5. Review Comments to the Author

Reviewer #1: Several studies published in the Literature so far reported a hypercoagulable profile in patients with acute COVID19-related pneumonia. However, there are still few studies that have tried to highlight a possible correlation between alteration of coagulation parameters and bad outcomes. The results of the study by Calvet et al. help bridge this gap. The study is clear and well written. The results are solid and the conclusions reported by the authors are in agreement with the data presented in the study. The only aspect on which I disagree is that of having associated the two end points "need for intubation" and "death in intensive care unit" in a single outcome. I believe that it is necessary to separately identify the coagulation profile that characterizes each of these two groups of patients and, consequently, to separately analyze the possible alterations of the coagulation profile for each of the two groups.

The request has been taken into account (see reply above to the Academic Editor)

Minor revisions: check the text for typos (i.e. abstract line 2 "thromboembolic (TE) events" change in "thromboembolic events (TE)".

All typos in the manuscript were corrected for thromboembolic (TE) events

Reviewer #2: The manuscript of Calvet et al. evaluates the coagulation profile according to both traditional coagulation tests and thromboelastometry parameters in a group of 133 patients admitted for acute COVID-19 pneumonia. Interestingly enough the authors tried to find a correlation between the alterations of coagulation parameters and a composite endpoint "intubation and / or death in intensive care unit". The study results demonstrate a possible association between hyperfibrinogenemia or fibrinolysis shutdown on day 4 and the composite outcome "intubation and / or death in intensive care unit".
The results presented in the paper support the study's conclusions. Statistical analysis is appropriate and rigorous.
My comments:
Abstract: "despite high use of therapeutic anticoagulation" I do not agree with this concept since both traditional coagulative parameters and ROTEM profiles are poorly influenced by low molecular weight heparin.

We understand your point and acknowledge that ROTEM tests are poorly influenced by LMWH. Consequently, we have removed this part of the sentence from the Abstract.

Introduction: the authors should explain in more detail the relationship between COVID-19 related pneumonia, hypercoagulability and hypofibrinolysis

Thank you for this excellent remark. We now added this sentence at the beginning of the introduction.

“Patients with severe forms of COVID 19 have multifactorial hypercoagulability: increased D-dimer, fibrinogen, factor VIII levels, decreased protein C, protein S and antithrombin levels, platelet hyperaggregability, endothelial damage by Sars Cov 2 [1–3], and hypofibrinolysis. This hypercoagulability leads to thromboembolic events (TE) concerning macro and micro circulation [4–7]. These TE events, notably microcirculation thrombosis and pulmonary embolism, are one of the leading causes of multiple organ failure in critically ill COVID-19 patients [4,6,8–14].”

Methods: the authors should better explain the principle of thromboelastometry and of the assays used.

Thank you for the comment. The request has been taken into account (see reply above to the Academic Editor)

Discussion: line 3 pag. 13 "Finally, we observed prolonged clot initiation on day 1 presumably owing to heparin exposure" see my comment above in the "abstract section".

Thank you for this excellent remark. The Discussion has been changed accordingly.

“In our study, we observed prolonged clot initiation. Whether it can be related to heparin exposure requires a careful analysis since the impact of heparin on INTEM-CT depends on both the type of heparin and its dosage. While ROTEM tests are able to detect the presence of UFH on the basis of an increase in the difference, or in the ratio, between INTEM-CT and HEPTEM-CT, and whereas in patients administered with UFH a prolonged INTEM-CT is reported for heparinemia > 0.1 U/l, both traditional coagulative parameters and ROTEM profiles are poorly influenced by LMWH, and an increased INTEM-CT in patients receiving LMWH is only observed for heparinemia >0.4UI/l[69,70]. We observed similar results in our study. INTEM/HEPTEM CT > 1 was detected in patients receiving UFH for HDPA or CDA, and in 90% of patients under LMWH for CDA, but only in half of the patients under LMWH for HDPA (S12 Table).”

Table S10: Anticoagulation and prolonged CT on day 4

| At day 4 | INTEM/HEPTEM CT < 1 | INTEM/HEPTEM CT > 1 | Pval |
| --- | --- | --- | --- |
| No anticoagulation | 2 (11.1) | 2 (4.5) | 0.02 |
| Preventive LMWH | 0 (0) | 1 (2.3) | . |
| LMWH HDPA | 13 (72.2) | 15 (34.1) | . |
| LMWH CDA | 2 (11.1) | 13 (29.5) | . |
| UFH HDPA | 0 (0) | 3 (6.8) | . |
| UFH CDA | 0 (0) | 10 (22.7) | . |
| Direct Oral Anticoagulants | 1 (5.6) | 0 (0) | . |
| Anti-vitamin K | 0 (0) | 0 (0) | . |

HDPA: high dose preventive anticoagulation; CDA: curative dose anticoagulation

6. PLOS authors have the option to publish the peer review history of their article. If published, this will include your full peer review and any attached files.


If you choose “no”, your identity will remain anonymous but your review may still be made public.


**Do you want your identity to be public for this peer review?** For information about this choice, including consent withdrawal, please see our [Privacy Policy](https://www.plos.org/privacy-policy).

Reviewer #1: No

Reviewer #2: No

[NOTE: If reviewer comments were submitted as an attachment file, they will be attached to this email and accessible via the submission site. Please log into your account, locate the manuscript record, and check for the action link "View Attachments". If this link does not appear, there are no attachment files.]

While revising your submission, please upload your figure files to the Preflight Analysis and Conversion Engine (PACE) digital diagnostic tool, <https://pacev2.apexcovantage.com/>. PACE helps ensure that figures meet PLOS requirements. To use PACE, you must first register as a user. Registration is free. Then, login and navigate to the UPLOAD tab, where you will find detailed instructions on how to use the tool. If you encounter any issues or have any questions when using PACE, please email PLOS at [figures@plos.org](mailto:figures@plos.org). Please note that Supporting Information files do not need this step.
